# Integrating fractional-order SEI1I2I3QCR model with awareness and non-pharmaceutical interventions for optimal COVID-19 pandemic

**DOI:** 10.1186/s12874-024-02452-7

**Published:** 2025-02-22

**Authors:** Ahmed Refaie Ali, Daniyal Ur Rehman, Najeeb Alam Khan, Muhammad Ayaz, Asmat Ara, M. Ijaz Khan

**Affiliations:** 1 Department of Mathematics and Computer Science, Faculty of Science, Menoufia University, Shebin El Kom, Menofia, 32511 Egypt; 2 Department of Computer Science, Iqra University, Karachi, PK 75300 Pakistan; 3 Department of Mathematics, University of Karachi, Karachi, 75270 Pakistan; 4 College of Humanities and Sciences, PAF-KIET, Karachi, 75190 Pakistan; 5 Department of Mechanics and Engineering Science, Peking University, Beijing, China; 6 Department of Mechanical Engineering, College of Engineering, Prince Mohammad bin Fahd University, Al-Khobar, Saudi Arabia

**Keywords:** Stability, Optimal control, SEI1I2I3QCR fractional derivative, Reproduction number, Numerical simulations COVID-19

## Abstract

Infectious diseases like COVID-19 continue to pose critical challenges globally, underscoring the need for effective control strategies that go beyond traditional vaccinations and treatments. This study introduces an advanced SEI1I2I3QCR model, uniquely incorporating fractional-order delay differential equations to account for latency periods and dynamic transmission patterns of COVID-19, improving accuracy in capturing disease progression and peak oscillations. Stability analyses of the model reveal the critical role of delay and fractional order parameters in managing disease dynamics. Additionally, we applied optimal control theory to simulate non-pharmaceutical interventions, such as quarantine and awareness campaigns, demonstrating a notable reduction in infection rates. Numerical simulations align the model closely with real-world COVID-19 data from China, validating its utility in guiding pandemic response strategies. Our findings emphasize the significance of integrating time-delay factors and fractional calculus in epidemic modeling, offering a novel framework for pandemic management through targeted, cost-effective control measures.

## Introduction

In the last two decades, more than thirty new diseases have emerged, posing a significant threat to the health of millions of people worldwide. As stated in a report by the World Health Organization (WHO), there is an imminent global epidemic of infectious diseases that cannot be ignored by any nation. Therefore, infectious diseases must be investigated and controlled. The control of such diseases has relied primarily on non-pharmaceutical interventions, such as quarantine, isolation, and social distancing. However, in the absence of timely detection of disease carriers, the accuracy of data in these centers becomes impaired. Consequently, prevention strategies are ineffective in reducing the transmission rate of infectious diseases. Various awareness campaigns are important in encouraging asymptomatic individuals to seek medical attention if they observe symptoms, rather than isolating themselves at home. In addition to medical researchers developing vaccines and antibiotics, mathematicians and epidemiologists also contribute to this effort.

The advancement in mathematical modeling techniques and the availability of computational power have been used by many scholars in the development of epidemic models [1, 2] that have gained significant attention. These models allow for a better understanding of Infectious diseases and the dynamics of their transmission [3] and can assist in the identification of critical parameters for the effective design of control strategies. Some epidemic models consist of quarantine, immunity [4–6] or vaccination [7] as an effective method for controlling the infectious disease spread. Investigating how a disease spreads from an infected individual to a healthy one is essential. The majority of the time, this mass action of transmission involves healthy people coming into contact with diseased people and then becoming infected themselves. Capasso and Serio [8] introduced saturated incidence rates. Recently [9–11] has used this type of incidence rate. Historically, SIR models based on ordinary differential equations have been used to analyze infectious disease spread [12, 13], as well as the cost control associated with different precautionary measures. In [14] an improved SEQIR model is proposed in order to predict the effects of control strategies used to minimize the incidence of infectious diseases in the population, and the effects of major interventions are examined [15] to estimate the transmission of disease. However, compared with ordinary differential equations it is not negligible to discuss the effect of the incubation period of time lag in the theory of epidemiological dynamical system [16, 17]. Due to the effect of the delay differential equation on infectious diseases spread in the population, there are various factors that contribute to their spread, such as delays associated with immunity periods and latency periods [18] etc. In [19] author proposes a delayed epidemic model in order to discuss the Hopf bifurcation with respect to the time lag that is significant for curing the infectious population as a parameter in bifurcation. Moreover, the integration of optimal control theory in epidemic modeling [20] can provide valuable insights into developing effective control strategies to reduce the transmission rate of pandemic diseases.

Fractional calculus has made significant advancements in recent years [21, 22]. Based on the definitions of Riemann–Liouville fractions and Caputo fractions, the literature contains many enhancements of these in several applications in the field of science such as physics and engineering [23, 24]. In [25] Caputo fractional order SIR epidemic model present with non-linear incidence rate. Recently, researchers in [26] have analyzed a non-linear fractional COVID-19 epidemic model. The interventions of COVID-19 model has been analyzed over transmissibility models. Undertaken the above applications of epidemiology and fractional derivative. In this paper, we aim to develop an epidemic model based on fractional order delay differential equations, incorporating optimal control theory to design effective control strategies for reducing the transmission rate of any pandemic disease that may appear in the population. By using our model and optimal control strategies, we hypothesize that we can significantly reduce the transmission rate of pandemic diseases, thereby improving the overall health and well-being of the population.

Here are explicit paragraphs to be added to the introduction section, enhancing its depth and context:

In recent years, infectious disease modeling has become increasingly vital for understanding and controlling the spread of pathogens, particularly in the context of global health crises like the COVID-19 pandemic. Traditional compartmental models, such as the SIR (Susceptible-Infectious-Recovered) framework, have provided foundational insights into disease dynamics; however, they often oversimplify complex transmission patterns by assuming immediate transitions between compartments and uniform population behavior. To address these limitations, researchers have turned to more sophisticated approaches that incorporate additional compartments and account for factors such as latency and symptom severity.

The SEI1I2I3QCR model presented in this study enhances the traditional SIR framework by introducing a fractional-order delay differential equation structure that accommodates the nuances of COVID-19 transmission. By segmenting the infectious population into asymptomatic (I1), mildly symptomatic (*I2*), and severely symptomatic (*I3*) categories, the model captures critical differences in infectiousness and treatment needs that influence overall disease dynamics. This compartmentalization allows for a more realistic representation of how individuals progress through different stages of infection, which is essential for designing effective control strategies.

Additionally, incorporating fractional-order calculus into the model offers significant advantages over conventional modeling techniques. Fractional-order derivatives account for memory effects and time delays, reflecting the reality that individuals do not transition instantaneously between states. This characteristic is particularly relevant in diseases like COVID-19, where the incubation period can result in a lag between infection and symptom onset, leading to complex transmission dynamics. By employing fractional-order delay differential equations, our model aims to provide a more accurate depiction of the pandemic's progression and the impact of non-pharmaceutical interventions (NPIs) such as quarantining and isolation.

Prior research has demonstrated the importance of symptom awareness in reducing the spread of infectious diseases. Increased public awareness regarding the recognition of symptoms can lead to earlier identification and isolation of infectious individuals, thereby limiting transmission within communities. This study investigates the role of awareness campaigns in enhancing compliance with preventive measures and aims to quantify their effectiveness within the SEI1I2I3QCR framework. By understanding the interplay between symptom awareness, compliance, and intervention strategies, we hope to contribute valuable insights for public health planning and response efforts. Recent studies have focused on the application of fractional-order models in epidemiology, highlighting their effectiveness in capturing the dynamics of disease spread and control. For instance, Atede et al. (2023) introduced a fractional-order vaccination model for COVID-19 that incorporates environmental transmission, providing valuable insights through a case study in Nigeria [27]. The use of Sumudu transform for solving fractional heat-like and wave-like equations has been demonstrated in several studies [28, 29], while fractional-order models have been applied to analyze dengue transmission dynamics [30] and assess intervention measures for co-dynamics of monkeypox and COVID-19 [31]. Similarly, Mangal et al. (2023) developed a fractional-order deterministic epidemic model for HIV/AIDS with a specific reference to Mexico and India, emphasizing the significance of tailored intervention strategies [32]. The work of Mangal and colleagues further extends to the modeling of SIRS epidemics using fractional-order differential equations, addressing factors such as the fear effect and public awareness [33]. Additionally, Bonyah et al. (2023) examined the social awareness perspective in fractional stochastic modeling of dengue fever [34], while Mangal et al. (2024) presented a novel fractional-order stochastic epidemic model to analyze the role of media awareness in the spread of conjunctivitis [35]. Furthermore, Mangal and collaborators explored the effects of vaccination in fractional-order epidemic models for various infectious diseases [36] and modeled the spread and control of multidrug-resistant tuberculosis in India [37]. These studies collectively underscore the growing importance of fractional modeling approaches in understanding and managing infectious diseases [27, 32–37].

Through this study, we aim to fill the existing gaps in the literature regarding the dynamics of COVID-19 transmission and the effectiveness of control measures. Our findings will provide a comprehensive understanding of how varying degrees of symptom awareness and compliance with NPIs can shape the trajectory of infectious disease outbreaks, informing strategies for future public health interventions.

In "Theoretical Analysis of Model" section, we develop the SEI1I2I3QCR model, focusing on its application for disease control and analyzing crucial characteristics such as existence, uniqueness, boundedness, and positivity within the differential equation framework. "Equilibrium states and stability analysis" and "Evaluation of endemic equilibrium structures and Hopf bifurcations" sections delve into the local stability analysis of equilibrium points, exploring both endemic and disease-free states. We also examine the conditions for Hopf bifurcation and provide insights into the basic reproduction number, which is vital for understanding disease dynamics. In "Formulation of Optimal Control Problem" section, we conduct an optimal control analysis to evaluate intervention strategies, while "Numerical Deliberation and Discussion" section presents numerical simulations alongside detailed discussions of the analytical results. This section also includes graphical representations that illustrate the model's behavior under various scenarios. Finally, "Important Discussion about the model" section offers a comprehensive summary of our findings, reinforcing the significance of our model in informing public health strategies and enhancing preparedness against infectious disease outbreaks.

## Theoretical analysis of model

The theoretical foundation of the SEI1I2I3QCR model is built upon the integration of fractional-order calculus and delay differential equations, which collectively enhance the model’s ability to simulate the dynamics of infectious disease transmission more accurately than traditional integer-order models. The use of fractional derivatives allows for the incorporation of memory effects, reflecting how past infections influence current disease states. This characteristic is particularly relevant in the context of COVID-19, where the dynamics of disease progression are influenced not only by the instantaneous rates of infection and recovery but also by the history of the population's exposure to the virus. Additionally, the model’s delay terms explicitly account for the incubation period, enabling a realistic representation of the time lag between exposure and infectiousness. This dual approach not only improves the accuracy of predictions but also provides insights into the potential oscillatory behaviors of disease transmission that may arise due to the interplay between infectious and susceptible individuals over time. The stability analysis conducted on this model further reveals critical insights into the conditions necessary for achieving disease-free equilibrium, as well as the thresholds at which interventions must be applied to prevent outbreaks. By employing advanced mathematical techniques to analyze the model’s behavior, we contribute a novel framework that enhances the understanding of how non-pharmaceutical interventions can effectively control the spread of infectious diseases, offering a template for future research on similar epidemiological challenges.

The use of mathematical models is crucial for understanding the dynamics of epidemics. By developing a model that is carefully designed, policymakers can foresee disease patterns and take the appropriate measures to prevent and control disease spread. Therefore, here we extended the classical SIR model [38] with consideration of stratified compartments in which infectious individuals were divided into three classes: asymptomatic, mildly symptomatic, and severely symptomatic. By analyzing the model mathematically, we investigate the dynamic behavior of infectious diseases and propose optimized control strategies based on a population N(t) divided into eight classes as follows: susceptible without a previous infection (seronegative S(t)), exposed E(t), Infected and pre-symptomatic (already infectious but not detected) I_1_(t), Infected with mild symptoms I_2_(t), Infected with severe symptoms I_3_(t), Quarantine Q(t), intensive care unit (I.C.U) C(t), Recovered R(t). Accordingly, the whole population N (t) at any given time t is represented as follows:

1 $$N(t) = \,S\left( t \right) + E\left( t \right) + I_{1} \left( t \right) + I_{2} \left( t \right) + I_{3} \left( t \right) + Q\left( t \right) + C\left( t \right) + R\left( t \right)$$

**Fig. 1:**
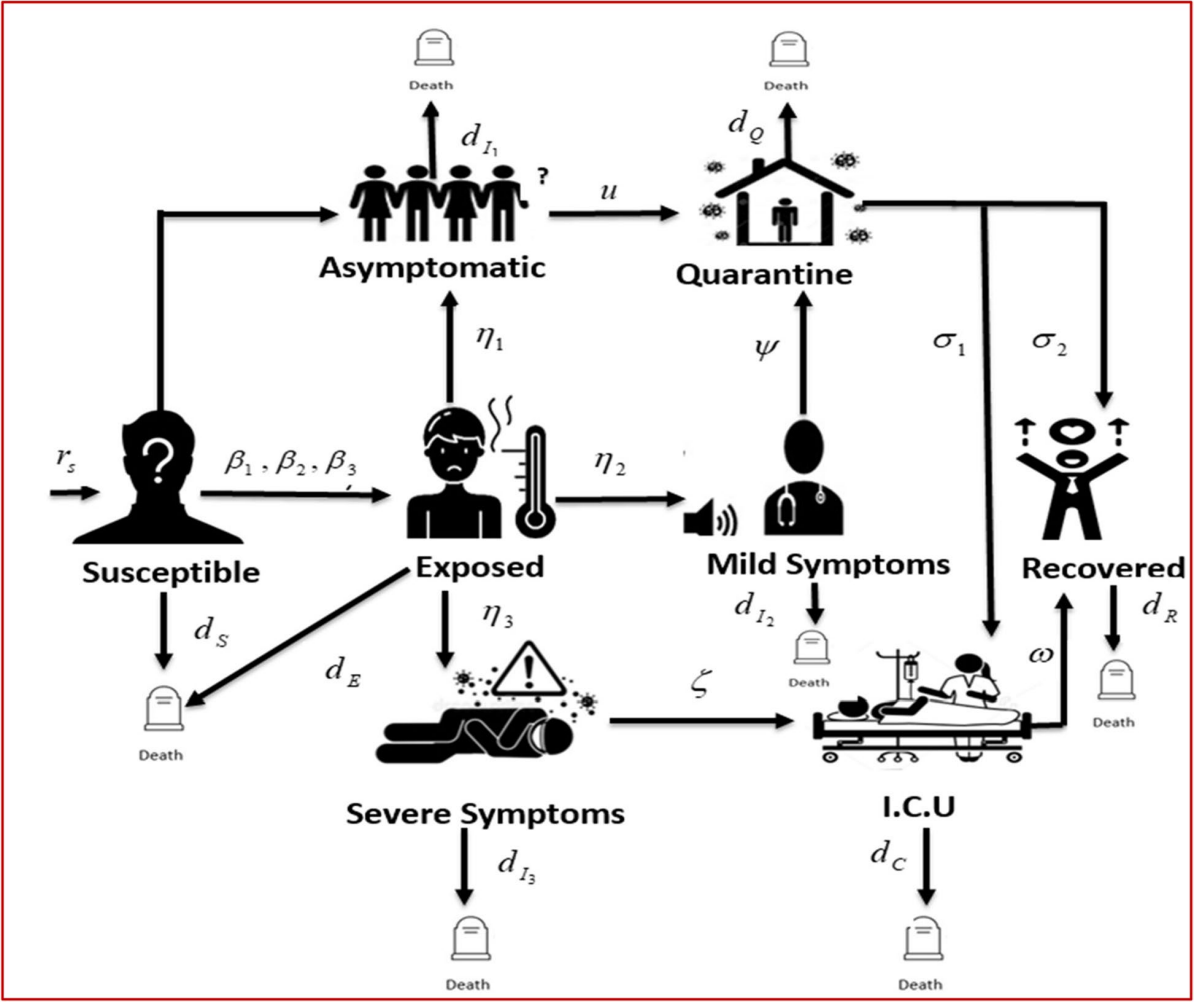
Schematic diagram of epidemic transmission model

**Fig. 2:**
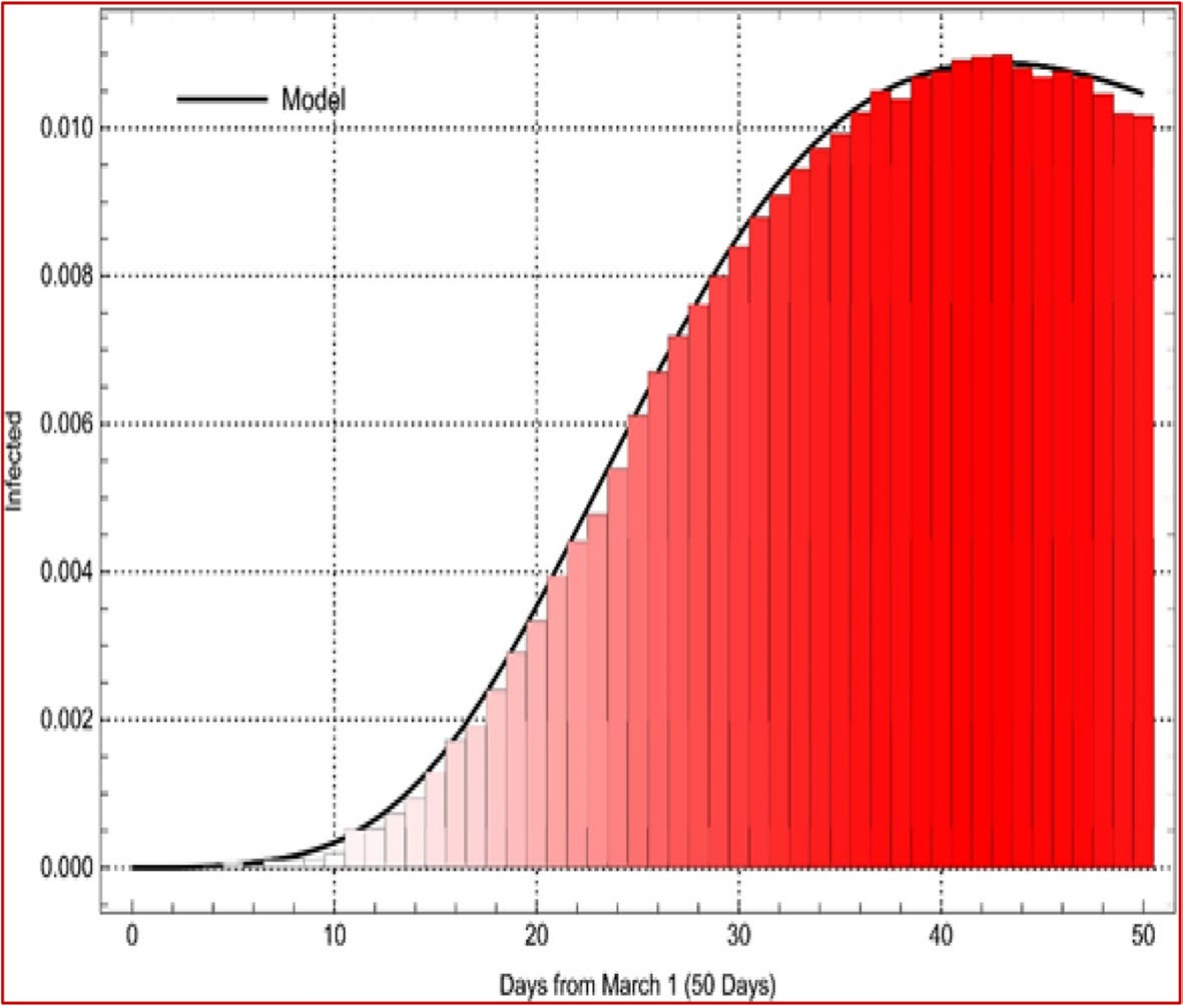
Data fitting by model (2) with daily cumulative cases of Covid-19

Graphically, the disease transmission circuit is shown in Figs. 1 and 2 using a compartmentalized epidemic model, we illustrate the classical path. Based on the following assumptions, a system of delay differential equations is developed to analyze the dynamics of pandemic disease and its prevention measures.

- Taking into account the varying risk rates of the pandemic disease among different age groups and individuals with pre-existing medical conditions, in the model, the assumption is that the population is homogeneous in terms of mixing of individuals.
- As a result of awareness campaigns conducted by the government or other healthcare organizations, infectious individuals become part of the Quarantine class after a period of time.
- In accordance with the operational prevention strategy, individuals in the asymptomatic compartment are assumed to be well informed after a certain period of time and are defined as being in quarantine.
- Logistic growth is evident in ensuring maximum sustainability in the environment in which resources are available.
- It is also generally assumed that individuals with mild symptoms of a disease should be placed in quarantine before completing their recovery.
- Recovery of severely infected patients is achieved by isolating them from their environment, which is explained in the ICU compartment. As long as the patients are receiving supportive care from the staff, they may be able to recover and move to the recovery compartment.
- When treated individuals use operational prevention measures after recovery, they are not linked to disease transmission.
- To evaluate reproduction numbers and conduct stability tests, a fractional calculus environment is used.

Thus, based on aforementioned assumptions, the fractional order epidemiological model SEI_1_I_2_I_3_QCR are mathematically demonstrated as:

2 $$\begin{array}{lllll}^{PF}D_t^\alpha S\left(t\right)=rS\left(t\right)\left(1-\frac{S(t)}k\right)-\left(\beta_1\,I_1\left(t\right)+\beta_2\,I_2\left(t\right)+\beta_3\,I_3\left(t\right)\right)S\left(t\right)-d_SS\left(t\right),\\{}^{PF}D_t^\alpha E\left(t\right)=\left(\beta_1\,I_1\left(t\right)+\beta_2\,I_2\left(t\right)+\beta_3\,I_3\left(t\right)\right)S\left(t\right)-\left(\eta_1+\eta_2+\eta_3+d_E\right)E\left(t\right),\\{}^{PF}D_t^\alpha I_1\left(t\right)=\eta_1\,E\left(t\right)-u\,I_1\left(t\right)-d_{I_1}\,I_1\left(t\right),\\{}^{PF}D_t^\alpha I_2\left(t\right)=\eta_2\,E\left(t\right)-\psi\,I_2\left(t\right)-d_{I_2}\,I_2\left(t\right),\\{}^{PF}D_t^\alpha I_3\left(t\right)=\eta_3\,E\left(t\right)-\xi\,I_3\left(t\right)-d_{I_3}\,I_3\left(t\right),\\{}^{PF}D_t^\alpha Q\left(t\right)=\psi\,I_2\left(t\right)-\sigma_1\,Q\left(t\right)-\sigma_2\,Q\left(t\right)+u\,I_1\left(t-\tau_1\right)-d_QQ\left(t\right),\\{}^{PF}D_{t}^{\alpha } C\left( t \right) = \sigma_{{_{1} }} \,Q\left( {t - \tau_{1} } \right) + \zeta I_{3} \left( t \right) - \omega C\left( t \right) - d_{C} C\left( t \right),\\{}^{PF}D_{t}^{\alpha } R\left( t \right) = \sigma_{{_{2} }} \,Q\left( {t - \tau_{2} } \right) + \omega C\left( {t - \tau_{2} } \right) - d_{R} R\left( t \right),\end{array}$$

Subjected to the positive initial conditions and history functions are assumed as:

3 $$\begin{array}{lllll}S\left(\vartheta\right)=\Omega_1\left(\upsilon\right),\quad E\left(\vartheta\right)=\Omega_2\left(\upsilon\right),\quad I_1\left(\vartheta\right)=\Omega_3\left(\upsilon\right),\quad I_2\left(\vartheta\right)=\Omega_4\left(\upsilon\right),\\I_3\left(\vartheta\right)=\Omega_5\left(\upsilon\right),\,\,\,Q\left(\vartheta\right)=\Omega_6\left(\upsilon\right),\quad C\left(\vartheta\right)=\Omega_7\left(\upsilon\right),\quad R\left(\vartheta\right)=\Omega_8\left(\upsilon\right)\end{array}$$

With, $\Omega_{i} \left( \upsilon \right) \ge 0$ and $\Omega_{1} \left( \upsilon \right)> 0$ for $i = 1,2, \ldots ,8$, such that $\left( {\Omega_{1} \left( \upsilon \right),\,\Omega_{2} \left( \upsilon \right),\, \cdots ,\,\Omega_{8} \left( \upsilon \right)} \right) \in \Phi \left( {\left[ { - \tau ,0} \right],\Re_{ + }^{8} } \right)$ where $\Phi \left( {\left[ { - \tau ,0} \right],\Re_{ + }^{8} } \right)$ be the Banach space of functions from $\left[ { - \tau ,0} \right]$ to $\Re_{ + }^{8}$. The parameters involved in system model (2) are describe in Table 1. As an application of proportional fractional derivatives [39]. A study of the dynamical behavior of the model is carried out at each fractional change in the system (2). After applying the derivatives, we can obtain these expressions for the governing model, which can be used for the simulation.

**Table 1:** Description of functions and parameters of model (2)

**State Variables**	**Descriptions**	**Estimated initial** **Values (population = '000 s & time = days)**		**Source**
		**Set 1**	**Set 2**	
*N*(*t*)	Total population	470	470	Estimated
*S*(*t*)	Susceptible	100	100	Estimated
*E*(*t*)	Exposed	0	0	Estimated
*I*_1_ (*t*)	Pre-symptomatic	100	100	Estimated
*I*_2_ (*t*)	Infected with mild symptoms	250	250	Estimated
*I*_3_ (*t*)	Infected with Severe symptoms	20	20	Estimated
*Q*(*t*)	Quarantined	0	0	Estimated
*C*(*t*)	Intensive Care unit	0	0	Estim ated
*R*(*t*)	Recovered	0	0	Estimated
**Parameters**	**Descriptions**	**Estimated transmission rates** **Individuals/(individuals × day)**		**Source**
		**Set 1**	**Set 2**	
*r*	Intrinsic growth rate of$S\left( t \right)$	500	500	[40]
*k*	Maximum sustainability of$S\left( t \right)$	1000	1000	Fitted
*β*_1_	Virus transmission rate of$S\left( t \right)$to$E\left( t \right)$after contacting$I_{1} \left( t \right)$	0.3781	0.781	Fitted
*β*_2_	Virus transmission rate of$S\left( t \right)$to$E\left( t \right)$after contacting$I_{1} \left( t \right)$	0.01781	0.023	Fitted
*β*_3_	Virus transmission rate of$S\left( t \right)$to$E\left( t \right)$after contacting$I_{1} \left( t \right)$	0.0378	0.612	Fitted
$\eta_{1}$	Rate of exposed to$I_{1} \left( t \right)$	0.471	0.771	Fitted
$\eta_{2}$	Rate of exposed to$I_{2} \left( t \right)$	0.127	0.771	Fitted
$\eta_{3}$	Rate of exposed to$I_{3} \left( t \right)$	0.0188	$1.887$	Fitted
$\psi$	Rate of infected to$Q\left( t \right)$	0.11362	0.11624	Fitted
$\xi$	Rate of Infected to$C\left( t \right)$	0.33042	0.33029	Fitted
$\sigma_1$	Rate of quarantined to$C\left( t \right)$	0.07214	0.0714	Fitted
$\sigma_2$	Rate of quarantined to$R\left( t \right)$	0.01782	1.382 × 10^–3^	Fitted
$\omega$	Rate of I.C.U to$R\left( t \right)$	0.052	0.013	Fitted
*d*_s_	$S\left( t \right)$death rate	0.15	0.15	Fitted
*d*_E_	$E\left( t \right)$death rate	0.844	0.823	Fitted
$d_{I_1}$	$I_{1} \left( t \right)$death rate	0.94	0.81	Fitted
$d_{I_2}$	$I_{2} \left( t \right)$death rate	0.824	0.84	[40]
$d_{I_3}$	$I_{3} \left( t \right)$death rate	0.17	0.17	[40]
*d*_Q_	$Q\left( t \right)$death rate	0.22	0.34	Fitted
*d*_C_	$C\left( t \right)$death rate	0.94	0.9	Fitted
*d*_R_	$R\left( t \right)$death rate	0.112	0.11	Fitted
*u*	Rate of infected individuals taking part in awareness campaign	0–1	0–1	Fitted

4 $$\begin{array}{lllll}\dot S\left(t\right)=\frac1\alpha\left(r\,S\left(t\right)\left(1-\frac{S\left(t\right)}k\right)-\left(\beta_1\,I_1\left(t\right)+\beta_2\,I_2\left(t\right)+\beta_3\,I_3\left(t\right)\right)S\left(t\right)-d_SS\left(t\right)-\left(1-\alpha\right)S\left(t\right)\right),\\\dot E\left(t\right)=\frac1\alpha\left(\left(\beta_1\,I_1\left(t\right)+\beta_2\,I_2\left(t\right)+\beta_3\,I_3\left(t\right)\right)S\left(t\right)-\left(\eta_1+\eta_2+\eta_3+d_E\right)E\left(t\right)-\left(1-\alpha\right)E\left(t\right)\right),\\{\dot I}_1\left(t\right)=\frac1\alpha\left(\eta_1E\left(t\right)-uI_1\left(t\right)-d_{I_1}I_1\left(t\right)-\left(1-\alpha\right)I_1\left(t\right)\right),\\{\dot I}_2\left(t\right)=\frac1\alpha\left(\eta_2\,E\left(t\right)-\left(\psi+d_{I_2}+\left(1-\alpha\right)\right)I_2\left(t\right)\right),\\{\dot I}_3\left(t\right)=\,\frac1\alpha\left(\eta_3E\left(t\right)-\left(\zeta+d_{I_3}+\left(1-\alpha\right)\right)\,I_3\left(t\right)\right),\\ \dot{Q}\left(t\right)=\frac1\alpha\,\left(\,\psi\,I_2\left(t\right)-\sigma_1\,Q\left(t\right)-\sigma_2\,Q\left(t\right)+u\,I_1\left(t-\tau_1\right)-d_QQ\left(t\right)-\left(1-\alpha\right)\,Q\left(t\right)\right),\\\dot{C}\left( t \right) = \frac{1}{\alpha }\left( {\sigma_{{_{1} }} \,Q\left( {t - \tau_{1} } \right) + \zeta I_{3} \left( t \right) - \omega C\left( t \right) - d_{C} C\left( t \right) - \left( {1 - \alpha } \right)I\left( t \right)} \right),\\\dot{R}\left( t \right) = \frac{1}{\alpha }\left( {\sigma_{{_{2} }} \,Q\left( {t - \tau_{2} } \right) + \omega \,C\left( {t - \tau_{2} } \right) - d_{R} R\left( t \right) - \left( {1 - \alpha } \right)R\left( t \right)} \right),\end{array}$$

The initial conditions specified in Eq. (3) will be maintained when performing further analysis. Using integer order system (4) to reduce the computationally complexities behind the solution of system (4) is equivalent to system (2). According to the elementary theory of ordinary differential equations [41], the system (4) will have positive solutions that are bounded, exist, and are unique, provided that the initial conditions are the same as defined in Eq. (2). These properties can also be inferred from [42].

**Theorem 1** *(Boundedness)*

$\Lambda \in \Re_{ + }^{8}$* is positive solutions of system (4) and bounded subset of *$\Re_{ + }^{8}$.

5 $$\Lambda = \left\{ {\left( {S,E,I_{1} ,I_{2} ,I_{3} ,Q,C,R} \right) \in \Re_{ + }^{8} \,;\,N \le \frac{r}{{\mu^{ * } k}}} \right\}$$

*Proof* The detailed proof is to be followed similarly as mentioned [43].

**Theorem 2** *(Existence and Uniqueness)*

*The matrix of the system (3) should be a real-valued function* $X\left( {{\rm Z}\left( t \right)} \right):\Re_{ + }^{8} \to \Re_{ + }^{8}$ *, such that* $X\left( {Z\left( t \right)} \right)$ *and* $\frac{{\partial X\left( {Z\left( t \right)} \right)}}{\partial Z\left( t \right)}$ *are continuous and*

6 $$\left\| {X\left( {Z\left( t \right)} \right),Z\left( {t - \tau_{1} } \right),Z\left( {t - \tau_{2} } \right)} \right\| \le \left( {\frac{\xi }{\left| \alpha \right|} - \tilde{\vartheta }} \right)\left\| {Z\left( t \right)} \right\| + \left\| {\left\| {A_{4} } \right\|\left\| {Z\left( {t - \tau_{1} } \right)} \right\| + \left\| {A_{5} } \right\|\left\| {Z\left( {t - \tau_{2} } \right)} \right\|} \right\| , \forall Z\left( t \right) \in \Re_{ + }^{8}\;and\;0 < \alpha \le 1$$

*Then, for a set of initial conditions that satisfy Eq. *(2)*, there exists a unique, positive, and bounded solution of the system (3) for all *$- \tau_{1} \le t \le \infty$* and *$- \tau_{2} \le t \le \infty$.

*Proof* The detailed proof is to be followed similarly as mentioned [43].

## Equilibrium states and stability analysis

In analyzing the equilibrium states and stability of the SEI1I2I3QCR model, we identify two primary equilibrium points: the disease-free equilibrium (DFE) and the endemic equilibrium (EE). The disease-free equilibrium represents a state in which the disease is no longer present in the population, while the endemic equilibrium reflects a persistent level of infection that remains in the community. A critical aspect of our analysis involves calculating the basic reproduction number (R_0​_), which serves as a threshold parameter determining the stability of these equilibria. Specifically, we demonstrate that when R_0_ < 1, the disease-free equilibrium is locally asymptotically stable, implying that the disease will die out under optimal conditions. Conversely, when R_0_ > 1, the model indicates that the disease will persist within the population, leading to an endemic state. This sensitivity to R_0​_ underscores the importance of non-pharmaceutical interventions, such as effective quarantine and awareness campaigns, in reducing transmission rates to achieve R_0​_ below the critical threshold. Furthermore, our stability analysis incorporates the effects of delay and fractional-order parameters, revealing that these factors introduce complex dynamics, including the potential for Hopf bifurcations. Such bifurcations signify transitions from stable to oscillatory behavior in disease transmission, reflecting real-world scenarios where outbreaks may exhibit periodic peaks. By exploring these equilibrium states and their stability conditions, we provide valuable insights into the dynamics of COVID-19 transmission and highlight the critical need for timely and effective public health interventions to manage the spread of infectious diseases effectively.

For the equilibrium points of system (4) we take.

$\dot{S}\left( t \right) = \dot{E}\left( t \right) = \dot{I}_{1} \left( t \right) = \dot{I}_{2} \left( t \right) = \dot{I}_{3} \left( t \right) = \dot{Q}\left( t \right) = \dot{C}\left( t \right) = \dot{R}\left( t \right) = 0$, The disease-free equilibrium points are always exist and denoted by $\Gamma_{1} \left( {S_{{_{0} }}^{*} ,0,0,0,0,0,0,0} \right)$, to check the local asymptomatic stability at DFE. We have the community matrix of system (4) at $\Gamma_{1}$ is given by.

$${J} = + e^{{-\lambda\tau_{1}}} + {C}e^{{-\lambda\tau_{2}}}$$, where $$X_{1} = \left( {\begin{array}{*{20}c} {a_{11} } & 0 & {a_{13} } & {a_{14} } & {a_{15} } & 0 & 0 & 0 \\ 0 & {a_{22} } & {a_{23} } & {a_{24} } & {a_{25} } & 0 & 0 & 0 \\ 0 & {a_{32} } & {a_{33} } & 0 & 0 & 0 & 0 & 0 \\ 0 & {a_{42} } & 0 & {a_{44} } & 0 & 0 & 0 & 0 \\ 0 & {a_{52} } & 0 & 0 & {a_{55} } & 0 & 0 & 0 \\ 0 & 0 & 0 & {a_{64} } & 0 & {a_{66} } & 0 & 0 \\ 0 & 0 & 0 & 0 & {a_{75} } & 0 & {a_{77} } & 0 \\ 0 & 0 & 0 & 0 & 0 & 0 & 0 & {a_{88} } \\ \end{array} } \right)$$ $$X_{2} = \left( {\begin{array}{*{20}c} {b_{63} } & {\mathbf{0}} & {\mathbf{0}} & {\mathbf{0}} \\ {\mathbf{0}} & {\mathbf{0}} & {\mathbf{0}} & {b_{76} } \\ \end{array} } \right)_{8 \times 8}$$ and $$X_{3} = \left( {\begin{array}{*{20}c} {\mathbf{0}} & {\mathbf{0}} \\ {c_{86} } & {c_{87} } \\ \end{array} \,\,} \right)_{8 \times 8}$$.

where, $$a_{11} = \frac{{1 - \alpha + d_{S} - r}}{\alpha }$$, $$a_{13} = \frac{{\left( {1 + d_{S} - r - \alpha } \right)\beta_{1} k}}{r\alpha }$$, $$a_{14} = \frac{{\left( {1 + d_{S} - r - \alpha } \right)\beta_{2} k}}{r\alpha }$$, $$a_{15} = \frac{{\left( {1 + d_{S} - r - \alpha } \right)\beta_{3} k}}{r\alpha }$$, $$a_{22} = \, \frac{{ - 1 - \alpha + d_{E} - \eta_{1} }}{\alpha }$$, $$a_{23} = \, \frac{{ - 1 - \alpha + d_{E} - \eta_{2} }}{\alpha }$$ $$a_{24} = \, \frac{{ - 1 - \alpha + d_{E} - \eta_{3} }}{\alpha }$$ $$a_{25} = \, \frac{{ - 1 - \alpha + d_{E} - \eta_{1} - \eta_{2} - \eta_{3} }}{\alpha }$$ $$a_{32} = \frac{{\eta_{1} }}{\alpha }$$ $$a_{33} = \, \frac{{ - 1 - d_{{I_{1} }} - u + \alpha }}{\alpha }$$, $$a_{42} = \, \frac{{\eta_{2} }}{\alpha }$$, $$a_{44} = \, \frac{{ - 1 - d_{{I_{2} }} + \alpha - \psi }}{\alpha }$$, $$a_{52} = \, \frac{{\eta_{3} }}{\alpha }$$, $$a_{55} = \, \frac{{ - 1 - d_{{I_{3} }} + \alpha - \theta }}{\alpha }$$, $$a_{64} = \, \frac{\psi }{\alpha }$$, $$a_{66} = \, \frac{{ - 1 - d_{Q} + \alpha - \gamma_{1} - \gamma_{2} }}{\alpha }$$, $$a_{75} = \, \frac{\theta }{\alpha }$$, $$a_{77} = \, \frac{{\left( { - 1 - d_{C} - \omega + \alpha } \right)}}{\alpha }$$, $$a_{86} = \, \frac{{\gamma_{2} }}{\alpha }$$, $$a_{88} = \, \frac{{\left( { - 1 - d_{R} + \alpha } \right)}}{\alpha }$$,

$$b_{63} = \, \frac{u}{\alpha }$$, $$b_{76} = \, \frac{{\sigma_{1} }}{\alpha }$$, $$c_{86} = \, \frac{{\sigma_{2} }}{\alpha }$$, $$c_{87} = \, \frac{{ - 1 - d_{R} + \alpha }}{\alpha }$$.

To get the stability analysis, we manipulate the Jacobian matrix $\text J$ of system (4) at $\Gamma_{1} \left( {S_{{_{0} }}^{*} ,0,0,0,0,0,0,0} \right)$ and obtain the three negative eigenvalues $\lambda$ i.e.$\lambda_{1} = a_{11}$, $\lambda_{2} = a_{22}$, $\lambda_{3} = a_{88}$, with the transcendental characteristic equation of $\text J$ as

7 $$\begin{array}{ll} \lambda^{5} + A_{4} \lambda^{4} + A_{3} \lambda^{3} + A_{2} \lambda^{2} + A_{1} \lambda + A_{0} + \left( {B_{4} \lambda^{4} + B_{3} \lambda^{3} + B_{2} \lambda^{3} + B_{1} \lambda + B_{0} } \right){\mathbf{e}}^{{{\mathbf{ - }}\lambda \tau_{1} }} \hfill \\ + \left( {C_{2} \lambda^{2} + C_{1} \lambda + C_{0} } \right){\mathbf{e}}^{{ - \lambda \tau_{2} }} + \left( {D_{1} \lambda + D_{0} } \right){\mathbf{e}}^{{{\mathbf{ - }}\lambda \left( {\tau_{1} + \tau_{2} } \right)}} = 0 \hfill \\ \end{array}$$

where the coefficients of $\lambda_{i}$ in (6) is given by:

$$\begin{array}{llll}A_{4} = { - }\left( {a_{{{22}}} + a_{{{33}}} + a_{{{44}}} + a_{{{55}}} + a_{{{77}}} } \right)\\A_{3} = \, a_{{{23}}} a_{32} + a_{{{24}}} a_{42} + a_{{{25}}} a_{52} - a_{{{44}}} a_{55} - \left( {a_{{{44}}} + a_{55} } \right)a_{{{77}}} - a_{{{33}}} \left( {a_{{{44}}} + a_{55} + a_{{{77}}} } \right) - a_{{{22}}} \left( {a_{{{33}}} + a_{44} + a_{{{55}}} + a_{77} } \right)\\A_{2} = \, a_{{{22}}} a_{33} a_{44} - a_{25} a_{33} a_{52} - a_{25} a_{33} a_{52} + a_{22} a_{33} a_{55} + a_{22} a_{44} a_{55} + a_{33} a_{44} a_{55} \hfill \\ + \left( {a_{{{33}}} a_{44} - a_{25} a_{52} + \left( {a_{33} + a_{44} } \right)a_{{{55}}} + a_{33} + a_{44} + a_{55} } \right)a_{77} - a_{24} a_{42} \left( {a_{33} + a_{55} + a_{77} } \right) - a_{23} a_{32} \left( {a_{44} + a_{55} + a_{77} } \right) \hfill\\A_{1} = - \, a_{{{23}}} a_{32} - a_{24} a_{42} - a_{25} a_{52} - a_{44} a_{55} - \left( {a_{44} + a_{55} } \right)a_{77} - \left( {a_{44} + a_{55} + a_{77} } \right)a_{33} - \left( {a_{33} + a_{44} + a_{55} + a_{77} } \right)a_{22}\end{array}$$

,

$$A_{0} = - \, \left( {a_{{{25}}} a_{33} a_{24} a_{52} + a_{24} a_{33} a_{42} a_{55} + a_{23} a_{32} a_{44} - a_{22} a_{33} a_{44} a_{55} } \right)a_{77}$$

$$B_{3} = { - }\left( {a_{33} + a_{55} + a_{7722} + a_{33} + a_{44} + a_{55} } \right)$$

$$B_{2} = \, \left( {a_{{{23}}} a_{32} + a_{{{24}}} a_{42} - a_{{{33}}} a_{44} - a_{{{25}}} a_{52} - \left( {a_{{{33}}} + a_{44} } \right)a_{{{55}}} - a_{{{22}}} \left( {a_{{{33}}} + a_{{{44}}} + a_{55} } \right)} \right)$$

$$B_{1} = \, \left( {a_{{{25}}} \left( {a_{33} + a_{44} } \right)a_{52} - a_{33} a_{44} a_{55} + a_{{{24}}} a_{42} \left( {a_{{{33}}} + a_{55} } \right)a_{{{55}}} + a_{{{23}}} a_{32} } \right)$$

$$B_{0} = \, \left( {a_{{{25}}} a_{33} a_{44} a_{52} + \left( {a_{{{24}}} a_{{{33}}} a_{{{42}}} + a_{23} a_{{{32}}} a_{{{44}}} - a_{{{23}}} a_{{{33}}} a_{{{44}}} } \right)a_{{{55}}} } \right)$$

$$C_{2} = \, a_{{{24}}} a_{52} b_{{{63}}}$$, $$C_{1} = \, a_{{{24}}} a_{52} \left( {a_{{{33}}} + a_{76} } \right)b_{{{63}}}$$, $$C_{0} = { - }a_{{{24}}} a_{33} a_{{{52}}} a_{77} a_{{{63}}}$$,

$$D_{1} = a_{63} a_{76} c_{86}$$, $$D_{0} = - b_{63} b_{76} c_{87}$$.

Here for the two time delays $\tau_{1}$ and $\tau_{2}$ we have the following theorems:

**Theorem 3** *For the time delay the disease-free equilibrium* $\Gamma_{1} \left( {S_{{_{0} }}^{*} ,0,0,0,0,0,0,0} \right)$ *is locally asymptotically stable for* $0 < \alpha \le 1$ *.*

*Proof* If $\tau_{1} = \tau_{2} = 0$ then Eq. (7) can be re-written as.

8 $$\lambda^{5} + \mu_{14} \lambda^{4} + \mu_{13} \lambda^{3} + \mu_{12} \lambda^{2} + \mu_{11} \lambda + \mu_{10} = 0$$

where,

$$\mu_{14} = A_{4}$$, $$\mu_{13} = A_{3} + B_{3}$$, $$\mu_{12} = A_{2} + B_{2} + C_{2}$$, $$\mu_{11} = A_{1} + B_{1} + C_{1} + D_{1}$$, $$\mu_{10} = A_{0} + B_{0} + C_{0} + D_{0}$$

Also we can easily see that

$$\mu_{14} = \frac{1}{\alpha }\left( {5 + d_{C} + d_{E} + d_{{I_{1} }} + d_{{I_{2} }} + d_{{I_{3} }} + u + \omega - 5\alpha + \eta_{1} + \eta_{2} + \eta_{1} + \rho + \sigma + \theta + \psi } \right)> 0$$

Then by Routh-Hurwitz criteria ${\text{Re}} \left( \lambda \right) < 0$, iff.

$$\mu_{10}> 0$$, $$\det \left( {\begin{array}{*{20}c} { \, \mu_{{{14}}} } & 0 \\ {\mu_{{{12}}} } & {\mu_{{{13}}} } \\ \end{array} } \right)> 0$$, $$\det \left( {\begin{array}{*{20}c} {\mu_{14} } & 1 & 0 \\ {\mu_{12} } & {\mu_{13} } & {\mu_{14} } \\ {\mu_{10} } & {\mu_{11} } & {\mu_{12} } \\ \end{array} } \right)> 0$$ and $$\text{det }\begin{pmatrix}\mu_{14}&1&0&0\\\mu_{12}&\mu_{13}&\mu_{14}&1\\\mu_{10}&\mu_{11}&\mu_{12}&\mu_{13}\\0&0&\mu_{10}&\mu_{11}\end{pmatrix}\text{>0}$$

Thus the DFE is locally asymptotically stable when $\tau_{1} = \tau_{2} = 0$.

**Theorem 4*** If Eq. *(12)* has a positive root then DFE *$\Gamma_{1} \left( {S_{{_{0} }}^{*} ,0,0,0,0,0,0,0} \right)$* is locally asymptotically stable when *$\tau_{1} = \left[ {0,\tau_{1}^{*} } \right)$*.*

*Proof *If $\tau_{1}> 0$ and $\tau_{2} = 0$ then Eq. (7) can be re-written as.

9 $$\lambda^{5} + \mu_{24} \lambda^{4} + \mu_{23} \lambda^{3} + \mu_{22} \lambda^{2} + \mu_{21} \lambda + \mu_{20} + \left( {\lambda^{4} + \chi_{23} \lambda^{3} + \chi_{22} \lambda^{2} + \chi_{21} \lambda + \chi_{20} } \right){\mathbf{e}}^{{ - \lambda \tau_{1} }} = 0$$

where

$$\mu_{24} = \mu_{14}$$, $$\mu_{23} = \mu_{13}$$, $$\mu_{22} = A_{2} + C_{2}$$, $$\mu_{21} = A_{1} + C_{1}$$, $$\mu_{20} = A_{0} + C_{0}$$, $$\chi_{23} = B_{3}$$, $$\chi_{22} = B_{2}$$, $$\chi_{21} = B_{1} + D_{1}$$, $$\chi_{20} = B_{0} + D_{0}$$.

Now suppose that, $\lambda = i\upupsilon \left( {\upupsilon> 0} \right)$ is a root of Eq. (8) then we have

10 $$g_{1} \sin \left( {\upupsilon \tau_{1} } \right) + g_{2} \cos \left( {\upupsilon \tau_{1} } \right) = h_{0}\;\text{and}\;g_{1} \cos \left( {\upupsilon \tau_{1} } \right) - g_{2} \sin \left( {\upupsilon \tau_{1} } \right) = h_{1}$$

Where.

$g_{1} = \chi_{23}\upupsilon ^{{3}} + \chi_{21}\upupsilon$, $g_{2} = \chi_{22} \lambda^{2} + \chi_{20}$, $h_{0} = - \mu_{24}\upupsilon ^{4} + \mu_{22}\upupsilon ^{2} - \mu_{20}$, $h_{1} = -\upupsilon ^{5} + \mu_{23}\upupsilon ^{3} - \mu_{21}\upupsilon$ so we can easily deduced that.

$g_{0}^{2} + g_{1}^{2} = h_{0}^{2} + h_{1}^{2}$ This implies that

11 $$\upupsilon ^{10} + \zeta_{24}\upupsilon ^{8} + \zeta_{23}\upupsilon ^{6} + \zeta_{22}\upupsilon ^{4} + \zeta_{21}\upupsilon ^{2} + \zeta_{20} = 0$$

where.

$$\zeta_{20} = \upsilon_{10}^{2} - \mu_{20}^{2}$$, $$\zeta_{21} = \mu_{21}^{2} + 2\mu_{22} \mu_{20} \chi_{23}$$, $$\zeta_{22} = - \mu_{22}^{2} - 2\mu_{21} \mu_{23} + 2\chi_{21} \chi_{20} - 2\mu_{20} \mu_{24} \,$$, $$\zeta_{23} = 2\mu_{21} + \mu_{23}^{2} + 2\mu_{22} \mu_{24} - 2\chi_{22}$$, $$\zeta_{24} = - 2\mu_{23} - \mu_{24}^{2}$$

We put $\upupsilon ^{2} = \kappa$ in (11) then we obtain

12 $$\kappa^{5} + \zeta_{24} \kappa^{4} + \zeta_{23} \kappa^{3} + \zeta_{22} \kappa^{2} + \zeta_{21} \kappa + \zeta_{20} = 0$$

If (12) has a positive root $\kappa_{1}^{*}$ then (11) has a positive root $\upupsilon = \sqrt {\kappa_{1}^{*} }$, now from the Eq. (10) we have the following condition

13 $$\cos \left( {\upupsilon \tau_{1}^{*} } \right) = \frac{{h_{0} + h_{1} }}{{g_{1} }} \Rightarrow \tau_{1}^{*} = \frac{1}{\upupsilon }\cos^{ - 1} \left( {\frac{{h_{0} + h_{1} }}{{g_{1} }}} \right)$$

Thus, Disease free equilibrium points is locally asymptotically stable when $\tau_{1}> 0$ and $\tau_{2} = 0$.

**Theorem 5*** If Eq. *(17)* has a positive root then DFE *$\Gamma_{1} \left( {S_{{_{0} }}^{*} ,0,0,0,0,0,0,0} \right)$* is locally asymptotically stable when *$\tau_{2} = \left[ {0,\tau_{2}^{*} } \right)$*.*

*Proof* If $\tau_{1} = 0$ and $\tau_{2}> 0$ then Eq. (7) can be re-written as.

14 $$\lambda^{5} + \mu_{34} \lambda^{4} + \mu_{33} \lambda^{3} + \mu_{32} \lambda^{2} + \mu_{31} \lambda + \mu_{30} + \left( {\chi_{32} \lambda^{2} + \chi_{31} \lambda + \chi_{30} } \right){\mathbf{e}}^{{ - \lambda \tau_{2} }} = 0$$

where.

$$\mu_{34} = A_{4}$$, $$\mu_{23} = A_{3} + B_{3}$$, $$\mu_{32} = A_{2} + B_{2}$$, $$\mu_{31} = A_{1} + B_{1}$$, $$\mu_{30} = A_{0} + B_{0}$$, $$\chi_{32} = C_{2}$$, $$\chi_{31} = C_{1} + D_{1}$$, $$\chi_{30} = C_{0} + D_{0}$$

Now, suppose that,$\lambda = i\ell \left( {\ell> 0} \right)$ is a root of Eq. (14) then we have

15 $$g_{2} \sin \left( {\ell \tau_{2} } \right) + g_{3} \cos \left( {\ell \tau_{2} } \right) = h_{2}\;\text{and}\;g_{2} \cos \left( {\ell \tau_{2} } \right) - g_{3} \sin \left( {\ell \tau_{2} } \right) = h_{3}$$

$g_{2} = \chi_{30} - \chi_{32} \ell^{2}$,$g_{3} = \chi_{31} \ell$,$h_{2} = - \mu_{34} \ell^{4} + \mu_{32} \ell^{2} - \mu_{30}$,$h_{3} = - \ell^{5} + \mu_{33} \ell^{3} - \mu_{31} \ell$ so we can easily deduced that

16 $$g_{2}^{2} + g_{3}^{2} = h_{2}^{2} + h_{3}^{2} \Rightarrow \ell^{10} + \zeta_{34} \ell^{8} + \zeta_{33} \ell^{6} + \zeta_{32} \ell^{4} + \zeta_{31} \ell^{2} + \zeta_{30} = 0$$

where,

$$\zeta_{30} = - \chi_{30}^{2} + \mu_{30}^{2}$$, $$\zeta_{31} = - \chi_{31}^{2} + 2\mu_{30} \chi_{32} + \mu_{31}^{2} - 2\mu_{30} \mu_{32}$$, $$\zeta_{32} = - \chi_{32}^{2} + \mu_{32}^{2} - 2\mu_{31} \chi_{33} + 2\mu_{30} \mu_{34}$$, $$\zeta_{33} = 2\mu_{31} + \mu_{33}^{2} - 2\mu_{32} \mu_{34}$$, $$\zeta_{34} = - 2\mu_{33} + \mu_{34}^{2}$$

Put $\ell^{2} = \wp$ in Eq. (16) then we obtain

17 $$\wp^{5} + \zeta_{34} \wp^{4} + \zeta_{33} \wp^{3} + \zeta_{32} \wp^{2} + \zeta_{31} \wp + \zeta_{30} = 0$$

If Eq. (17) has a positive root $\wp^{*}$ then Eq. (16) has a positive root $\ell = \sqrt {\wp^{*} }$, now from the Eq. (15). We have the following condition

18 $$\cos \left( {\omega \tau_{2}^{*} } \right) = \frac{{g_{2} h_{2} + g_{3} h_{3} }}{{g_{2}^{2} + g_{3}^{2} }} \Rightarrow \tau_{2}^{*} = \frac{1}{\ell }\cos^{ - 1} \left( {\frac{{g_{2} h_{2} + g_{3} h_{3} }}{{g_{2}^{2} + g_{3}^{2} }}} \right)$$

Thus, Disease free equilibrium points is locally asymptotically stable when $\tau_{1} = 0$ and $\tau_{2}> 0$

**Theorem 6 ***If Eq. *(21)* has a positive root then DFE *$\Gamma_{1} \left( {S_{{_{0} }}^{*} ,0,0,0,0,0,0,0} \right)$* is locally asymptotically stable when *$\tau = \left[ {0,\tau^{*} } \right)$

*Proof* If $\tau_{1} = \tau_{2} = \tau> 0$ then Eq. (7) can be re-written as.

19 $$\begin{gathered} \left( {\lambda^{5} + \mu_{44} \lambda^{4} + \mu_{43} \lambda^{3} + \mu_{42} \lambda^{2} + \mu_{41} \lambda + \mu_{40} } \right){\mathbf{e}}^{\lambda \tau } \hfill \\ \,\,\,\,\,\,\,\,\,\,\,\,\,\,\,\,\,\,\,\,\,\,\,\,\,\,\,\,\,\,\,\,\, + \chi_{44} \lambda^{4} + \chi_{43} \lambda^{3} + \chi_{42} \lambda^{2} + \chi_{41} \lambda + \chi_{40} + \left( {q_{41} \lambda + q_{40} } \right){\mathbf{e}}^{ - \lambda \tau } = 0 \hfill \\ \end{gathered}$$

where,

$$\mu_{44} = A_{4}$$, $$\mu_{43} = A_{3}$$, $$\mu_{42} = A_{2}$$, $$\mu_{41} = A_{1}$$, $$\mu_{40} = A_{0}$$, $$\chi_{44} = B_{4}$$, $$X_{43}=B_{3}$$, $$\chi_{42} = B_{2} + C_{2}$$, $$\chi_{41} = B_{1} + C_{1}$$, $$\chi_{40} = B_{0} + C_{0}$$, $$q_{41} = D_{1}$$, $$q_{40} = D_{2}$$

Now suppose that, $\lambda = i\nu \left( {\nu> 0} \right)$ is a root of Eq. (19) then we have

20 $$g_{4} {\text{Cos}} \left( {\nu \tau } \right) - g_{5} \sin \left( {\nu \tau } \right) = h_{4}\;\text{and}\;g_{4}^{*} \sin \left( {\nu \tau } \right) + g_{5}^{*} \cos \left( {\nu \tau } \right) = h_{5}$$

With

$$\begin{array}{ll}g_{4} = \mu_{44} \nu^{4} - \mu_{42} \nu^{2} + \chi_{40} + \upsilon_{40} + q_{40}, g_{5} = - \mu_{44} \nu^{4} + \mu_{42} \nu^{2} - \chi_{40} - \upsilon_{40} + q_{41},\\h_{4} = \chi_{42} \nu^{2} - \chi_{40} , g_{{_{4} }}^{*} = \nu^{5} + \mu_{41} \nu - \mu_{43} \nu^{3} , g_{{_{5} }}^{*} = - \nu^{5} - \mu_{41} \nu + \mu_{43} \nu^{3} , h_{5} = \chi_{40} \nu^{3}\end{array}$$

so we can easily deduce that

$$\cos \left( {\nu \tau } \right) = \frac{{\hbar_{1} }}{h},\sin \left( {\nu \tau } \right) = \frac{{\hbar_{2} }}{h}$$, where,

$$\begin{array}{lll}\hbar_{1} = \chi_{42} \nu^{7} + \chi_{41} \nu^{6} - \left( {\chi_{40} + \mu_{43} + q_{40} } \right)\nu^{5} - \chi_{41} \mu_{43} \nu^{4} + \left( {\mu_{43} \chi_{40} + \mu_{43} \chi_{42} } \right)\nu^{3} - \chi_{40} \mu_{41} \nu\\\hbar_{2} = \chi_{42} \nu^{7} - \left( {\chi_{40} - \mu_{44} \chi_{41} + \mu_{43} \chi_{42} } \right)\nu^{5} + \left( {\mu_{43} \chi_{40} - \mu_{42} \chi_{41} + \mu_{41} \chi_{42} } \right)\nu^{3} + \left( {\mu_{40} \chi_{41} - \mu_{41} \chi_{40} + \chi_{41} q_{40} } \right)\nu,\\h = 2\mu_{40} \nu^{9} - \left( {2\mu_{42} + 2\mu_{43} \mu_{44} } \right)\nu^{7} + 2\left( {\mu_{40} + \mu_{42} \mu_{43} + \mu_{41} \mu_{44} + q_{41} } \right)\nu^{5} - 2\left( {\mu_{41} \mu_{42} + \mu_{40} \mu_{43} + \mu_{43} q_{40} } \right)\nu^{3} + 2\left( {\mu_{40} \mu_{4!} + \mu_{41} q_{40} } \right)\nu^{7}\end{array}$$

Furthermore,

21 $$\hbar_{1}^{2} + \hbar_{2}^{2} = h^{2}$$

Hence, from Eq. (20) we have

22 $$\cos \left( {\omega_{0} \tau^{*} } \right) = \frac{{\hbar_{1} }}{h} \Rightarrow \tau^{*} = \frac{1}{{\omega_{0} }}\cos^{ - 1} \left( {\frac{{\hbar_{1} }}{h}} \right)$$

Thus, The DFE $\Gamma_{1}$ is locally asymptotically stable when $\tau_{1} = \tau_{2} = \tau> 0$.

## Evaluation of endemic equilibrium structures and Hopf bifurcations

The evaluation of endemic equilibrium structures and the analysis of Hopf bifurcations are critical components of understanding the long-term dynamics of the SEI1I2I3QCR model. At the endemic equilibrium, the model reveals a stable state where the infection persists within the population, governed by the balance between new infections and recoveries. Our analysis indicates that the existence of multiple endemic equilibria may arise depending on the values of transmission rates, recovery rates, and the effectiveness of non-pharmaceutical interventions. This multiplicity highlights the importance of accurately estimating these parameters to predict potential outcomes of intervention strategies.

Moreover, the analysis of Hopf bifurcations introduces the possibility of oscillatory dynamics in disease transmission, which can occur when the system transitions from stable to periodic behavior as certain critical parameters are varied. This phenomenon is particularly relevant in real-world scenarios where outbreaks may exhibit cyclical patterns due to seasonal effects or periodic intervention strategies. By identifying the parameter thresholds that lead to Hopf bifurcations, our model provides critical insights into when and how interventions should be adjusted to prevent oscillations that could lead to resurgence in infection rates. This understanding is vital for policymakers aiming to maintain control over infectious diseases in dynamic environments. Overall, the exploration of endemic equilibria and Hopf bifurcations within the SEI1I2I3QCR model not only enhances our theoretical understanding of disease dynamics but also informs practical strategies for managing public health responses during fluctuating epidemic conditions.

In this section, we use the analysis of [17] and find that system (4) exhibits local stability at a unique endemic equilibrium and Hopf bifurcation at a unique endemic equilibrium $\Gamma_{2} \left( {S^{*} ,E^{*} ,I_{1}^{*} ,I_{2}^{*} ,I_{3}^{*} ,Q^{*} ,C^{*} ,R^{*} } \right) \in \Re_{ + }^{8}$. As part of our stability analysis, we use the reproduction number $R_{o}$ for system (4), we employ the next-generation method [44]. A sub-model of SEI_1_I_2_I_3_QCR (3) is considered for this method, which consists only of infectious compartments which contains the exposed, pre-symptomatic, symptomatic, severe infected persons, quarantine, and infected individuals in intensive care unit (I.C.U). Therefore, following the method described in [42] we are able to obtain the basic reproduction number $R_{o}$, by taking the spectral radius ${\mathbf{K}}\left( {{\mathbf{FV}}^{{{\mathbf{ - 1}}}} } \right)$ of next generation matrix for system (4) and it follows as:

23 $$\text{R}_{\text{o}} = \frac{{ka_{11} \left( {\beta_{1} \eta_{1} a_{44} a_{55} + \beta_{2} \eta_{2} a_{33} a_{55} + \beta_{3} \eta_{3} a_{44} } \right)}}{{ra_{22} a_{33} a_{44} a_{55} }}$$

Next, we consider the community matrix of system (4) to analyses the local stability at $\Gamma_{2} \left( {S^{*} ,E^{*} ,I_{1}^{*} ,I_{2}^{*} ,I_{3}^{*} ,Q^{*} ,C^{*} ,R^{*} } \right) \in \Re_{ + }^{8}$ and obtain the negative eigenvalues,

$$\lambda_{1} = - 1 + d_{S} + \frac{{\beta_{1} \Lambda_{1} }}{{\Lambda_{2} }} - \frac{r}{\alpha }$$,$$\lambda_{2} = \frac{{ - 1 + d_{R} + \alpha }}{\alpha }$$, $$\lambda_{2} = \frac{{ - 1 - \alpha + d_{E} - \eta_{1} - \eta_{2} - \eta_{3} }}{\alpha }$$.

In the linearized system (4), the characteristic equation at $\Gamma_{2}$ is as follows:

24 $$\begin{gathered} \lambda^{5} + T_{4} \lambda^{4} + T_{3} \lambda^{3} + T_{2} \lambda^{2} + T_{1} \lambda + T_{0} + \left( {U_{3} \lambda^{3} + U_{2} \lambda^{3} + U_{1} \lambda + U_{0} } \right){\mathbf{e}}^{{{\mathbf{ - }}\lambda \tau_{1} }} \hfill \\ \,\,\,\,\,\,\,\,\,\,\,\,\,\,\,\,\,\,\,\,\,\,\,\,\,\,\,\,\,\,\,\,\,\,\,\,\,\,\,\,\,\,\,\,\,\,\,\,\,\,\,\,\,\,\,\,\,\,\,\,\,\,\,\,\,\,\,\,\,\, + \left( {V_{2} \lambda^{2} + V_{1} \lambda + V_{0} } \right){\mathbf{e}}^{{ - \lambda \tau_{2} }} + \left( {W_{1} \lambda + W_{0} } \right){\mathbf{e}}^{{{\mathbf{ - }}\lambda \left( {\tau_{1} + \tau_{2} } \right)}} = 0 \hfill \\ \end{gathered}$$

where, the expanded form of $$T_{i}$$, $$U_{i} ,$$$$V_{i}$$ and $$W_{i} ,$$ for $$i=0,1,2,3,4$$ are given in Appendix.

If $\tau_{1} = \tau_{1} = 0$, then Eq. (24) can be re-written as:

25 $$\lambda^{5} + {\varvec{K}}_{14} \lambda^{4} + {\varvec{K}}_{13} \lambda^{3} + {\varvec{K}}_{12} \lambda^{2} + {\varvec{K}}_{11} \lambda + {\varvec{K}}_{10} = 0$$

With

$$\boldsymbol K_{14}=T_{4}, \boldsymbol K_{13}=T_{3}+U_{3},\boldsymbol K_{12}=T_{2}+U_{2}+V_{2},\boldsymbol K_{11}=T_{1}+U_{1}+W_{1},\boldsymbol K_{10}=T_{0}+U_{0}+W_{0},$$

It is obvious that $\textbf{K}_{14}> 0$, As a result, applying the Routh-Hurwitz criteria leads, if and only if.

$${\varvec{K}}_{10}> 0$$, $$\det_{2} \left( {\begin{array}{*{20}c} { \, {\varvec{K}}_{{{14}}} } & 0 \\ {{\varvec{K}}_{{{12}}} } & {{\varvec{K}}_{{{13}}} } \\ \end{array} } \right)> 0$$, $$\det_{3} \left( {\begin{array}{*{20}c} {{\varvec{K}}_{14} } & 1 & 0 \\ {{\varvec{K}}_{12} } & {{\varvec{K}}_{13} } & {{\varvec{K}}_{14} } \\ {{\varvec{K}}_{10} } & {{\varvec{K}}_{11} } & {{\varvec{K}}_{12} } \\ \end{array} } \right)> 0$$ and $$\det_{4} \left( {\begin{array}{*{20}c} {{\varvec{K}}_{14} } & 1 & 0 & 0 \\ {{\varvec{K}}_{12} } & {{\varvec{K}}_{13} } & {{\varvec{K}}_{14} } & 1 \\ {{\varvec{K}}_{10} } & {{\varvec{K}}_{11} } & {{\varvec{K}}_{12} } & {{\varvec{K}}_{13} } \\ 0 & 0 & {{\varvec{K}}_{10} } & {{\varvec{K}}_{11} } \\ \end{array} } \right)> 0$$.

Hence, $\Gamma_{2} \in \Re_{ + }^{8}$ is locally asymptotically stable if $\tau_{1} = \tau_{2} = 0$. Furthermore, for different cases of delays, $\tau_{1}$ and $\tau_{2}$, we have the following theorems.

**Theorem 7** *The endemic equilibrium points* $\Gamma_{2} \left( {S^{*} ,E^{*} ,I_{1}^{*} ,I_{2}^{*} ,I_{3}^{*} ,Q^{*} ,C^{*} ,R^{*} } \right) \in \Re_{ + }^{8}$ *is Local asymptotically stable for the following cases.*

i) Local asymptotically stable for $\tau_{1}> 0$,$\tau_{2} = 0$, then $\Gamma_{2} \in \Re_{ + }^{8}$ when $\tau_{1} \in \left[ {0,\tau_{10} } \right)$; model (4) undergoes a Hopf bifurcation at $\Gamma_{2}$ when $\tau_{1} = \tau_{10}$.
ii) Local asymptotically stable for $\tau_{1} = 0$, $\tau_{2}> 0$, $\Gamma_{2} \in \Re_{ + }^{8}$ when $\tau_{2} \in \left[ {0,\tau_{20} } \right)$; model (4) undergoes a Hopf bifurcation at $\Pi_{2}$ when $\tau_{2} = \tau_{20}$.
iii) If $\tau_{1} = \tau_{2} = \tau> 0 \,$, $\Gamma_{2} \in \Re_{ + }^{8}$ the model (4) is locally asymptotically stable when $\tau \in \left[ {0,\tau_{0} } \right)$; model (4) undergoes a Hopf bifurcation at $\Gamma_{2}$ when $\tau = \tau_{0}$.

The proof is followed similarly as mentioned in [5, 7].

## Formulation of optimal control problem

The formulation of the optimal control problem within the SEI1I2I3QCR model is a critical advancement that allows for the systematic evaluation of intervention strategies aimed at minimizing the spread of COVID-19 while balancing associated costs. In this framework, we define a cost function that encompasses the financial implications of quarantine measures, awareness campaigns, and healthcare expenditures related to treating infected individuals. The objective is to determine the optimal levels of control measures, specifically the rates of quarantine for pre-symptomatic (I1) and mild cases (I2) as well as the intensity of awareness campaigns directed at the susceptible population.

To achieve this, we employ Pontryagin's Maximum Principle, which provides necessary conditions for optimality by deriving a set of adjoint equations that facilitate the evaluation of the optimal control strategies over a defined time horizon. The model incorporates dynamic feedback, allowing for real-time adjustments to the control parameters based on current epidemic conditions. By simulating various scenarios, we assess how different intensities of intervention can effectively reduce the reproduction number (R0) and lower infection rates.

Our analysis reveals that a well-structured optimal control strategy not only minimizes direct healthcare costs but also curtails the overall economic impact of the pandemic by preventing large outbreaks that would strain healthcare systems and disrupt social activities. Moreover, the model demonstrates that early and aggressive implementation of awareness campaigns can significantly enhance the effectiveness of quarantine measures, leading to a more sustainable public health response. The results from the optimal control problem formulation highlight the importance of integrating economic considerations into public health planning, ultimately providing a robust framework for decision-makers to allocate resources effectively during an ongoing epidemic.

The concept of optimal control seems to be the best mathematical approach to be used to address the issues related to deploying the best choice to achieve a certain goal. The theory of Pontryagin [20, 42] has been implemented to maximize the efficiency of upcoming decisions, A number of integer and fractional order epidemic models have been subjected to optimal control problems. This paper illustrates the state of control for SEI_1_I_2_I_3_QCR using the optimal control approach. By optimizing the control of the model (4), the cost of available treatment strategies can be minimized It is evident from Theorem 2 that the model (4) possesses a unique solution.

### Cost function

Here, we design an effective control plan for recognizing and reducing the number of infected people in the pre-symptomatic compartment. Furthermore, we reduce the potential intervention expenses that may arise from utilizing electronic and social media platforms for awareness campaigns. Assuming that the infected population becomes conscious at a rate of $u(t)$, we define the optimum control issue using the cost function as follows, taking into account the constant controlling parameter throughout the process:

26 $$\min \,J\mathop {(\theta_{i} ,u)}\limits_{u \in U} = \int\limits_{0}^{{t_{f} }} {\sum\limits_{i = 1}^{8} {\vartheta_{i} \theta_{i}^{2} + \Phi u^{2} } }$$

Here, $\Phi$ are the weight of intervention cost came in the spread of disease transmission and $\theta_{i}$ for $i = 1,2,3...8$, are replaced by the state functions $S,E,I_{1} ,I_{2} ,I_{3} ,Q,C,R,$ respectively. The expense of government awareness efforts or supportive treatment for patients in the ICU compartment rises with the number of infected persons. A rise in infection rates is the cause of this. We assume $\Phi> 0$. In the present scenario there is a need to control the spread of pandemic illnesses into the population as well as to minimize the number of people who become infected. Therefore, we consider $\vartheta_{i}> 0$ for $i = 3,4,5.$, and remaining equal to zero.

### Existence and necessary condition

A Hamiltonian function is explained in this section [19] Here we explains how to formulate a Hamiltonian function that is utilized to obtain the optimality requirements needed for the optimal control system (4). In order to optimize the solution, we first define the Lagrangian by:

27 $$L\left( {\theta_{i} } \right) = \sum\limits_{i = 1}^{8} {\vartheta_{i} \theta_{i}^{2} + \Phi u^{2} }$$

To achieve the minimum Lagrangian value, we set.

$$\theta \left( t \right) = \left( {S\left( t \right),E\left( t \right),I_{1} \left( t \right),I_{2} \left( t \right),I_{3} \left( t \right),Q\left( t \right),C\left( t \right),R\left( t \right)} \right)$$, $$\theta_{\tau_{1}} \left( t \right) = \left( S_{\tau_{1}} \left( t \right),E_{\tau_{1}} \left( t \right),I_{1 \tau_{1}} \left( t \right),I_{2 \tau_{1}} \left( t \right),I_{3 \tau_{1}} \left( t \right),Q_{\tau_{1}} \left( t \right),C_{\tau_{1}} \left( t \right),R_{\tau_{1}} \left( t \right) \right)$$.

and $$\theta_{\tau_{2}} \left( t \right) = \left( S_{\tau_{2}} \left( t \right),E_{\tau_{2}} \left( t \right),I_{1 \tau_{2}} \left( t \right),I_{2 \tau_{2}} \left( t \right),I_{3 \tau_{2}} \left( t \right),Q_{\tau_{2}} \left( t \right),C_{\tau_{2}} \left( t \right),R_{\tau_{2}} \left( t \right) \right)$$.

Also,

$$\begin{gathered} S_{\tau_{1}} \left( t \right) = S\left( t - \tau_{1} \right),E_{\tau_{1}} \left( t \right) = E\left( t - \tau_{1} \right),I_{1 \tau_{1}} \left( t \right) = I_{1} \left( t - \tau_{1} \right),I_{2 \tau_{1}} \left( t \right) = I_{2} \left( t - \tau_{1} \right), \hfill \\ I_{3 \tau_{1}} \left( t \right) = I_{3} \left( t - \tau_{1} \right),Q_{\tau_{1}} \left( t \right) = Q\left( t - \tau_{1} \right),C_{\tau_{1}} \left( t \right) = C\left( t - \tau_{1} \right),R_{\tau_{1}} \left( t \right) = R\left( t - \tau_{1} \right). \hfill \\ \end{gathered}$$

and

$$\begin{gathered} S_{\tau_{2}} \left( t \right) = S\left( t - \tau_{2} \right),E_{\tau_{2}} \left( t \right) = E\left( t - \tau_{2} \right),I_{1 \tau_{2}} \left( t \right) = I_{1} \left( t - \tau_{2} \right),I_{2 \tau_{2}} \left( t \right) = I_{2} \left( t - \tau_{2} \right), \hfill \\ I_{3 \tau_{2}} \left( t \right) = I_{3} \left( t - \tau_{2} \right),Q_{\tau_{2}} \left( t \right) = Q\left( t - \tau_{2} \right),C_{\tau_{2}} \left( t \right) = C\left( t - \tau_{2} \right),R_{\tau_{2}} \left( t \right) = R\left( t - \tau_{2} \right). \hfill \\ \end{gathered}$$

We also define $u_{{\tau_{1} }} = u\left( {t - \tau_{1} } \right)$ and $u_{{\tau_{2} }} = u\left( {t - \tau_{2} } \right)$. The Hamiltonian ${\mathbf{H}}$ is defined as the inner product of the right-hand side of the state model (4), as well as the adjoint variables are also described as $\Omega = \left( {\xi_{1} ,\xi_{2} ,\xi_{3} ,\xi_{4} ,\xi_{5} ,\xi_{6} ,\xi_{7} ,\xi_{8} } \right)$. Thus, we get

28 $$\begin{gathered} {\mathbf{H}}\left( {\theta ,\theta_{{\tau_{1} }} ,\theta_{{\tau_{2} }} ,u,u_{{\tau_{1} }} ,u_{{\tau_{1} }} ,\Omega } \right) = {\text{L}} \left( {\theta {}_{i}} \right) + \xi_{1} \left( t \right)\dot{S}\left( t \right) + \xi_{2} \left( t \right)\dot{E}\left( t \right) + \xi_{3} \left( t \right)\dot{I}_{1} \left( t \right) + \xi_{4} \left( t \right)\dot{I}_{2} \left( t \right) \hfill \\ \,\,\,\,\,\,\,\,\,\,\,\,\,\,\,\,\,\,\,\,\,\,\,\,\,\,\,\,\,\,\,\,\,\,\,\,\,\,\,\,\,\,\,\,\,\,\,\,\,\,\,\,\,\,\,\,\,\,\,\,\,\,\,\,\,\,\,\,\,\,\,\,\,\,\,\,\,\,\,\,\,\,\,\,\,\,\,\, + \xi_{5} \left( t \right)\dot{I}_{3} \left( t \right) + \xi_{6} \left( t \right)\dot{Q}\left( t \right) + \xi_{7} \left( t \right)\dot{C}\left( t \right) + \xi_{8} \left( t \right)\dot{R}\left( t \right) \hfill \\ \end{gathered}$$

We now proceed to determine the variable $\Omega$, Subsequently, we employ Pontryagin's maximum principle [5, 7] to construct the adjoint variables for the Hamiltonian, as defined in the following theorem.

**Theorem 8** *The Controlling function* $u^{ * }$ *together with the solution,*

$\left( {S^{*} \left( t \right),E^{*} \left( t \right),I_{1}^{*} \left( t \right),I_{2}^{*} \left( t \right),I_{3}^{*} \left( t \right),Q^{*} \left( t \right),C^{*} \left( t \right),R^{*} \left( t \right)} \right)$ *, of the optimal trajectory of the system (4), we can derive the corresponding adjoint variables,* $\Omega = \left( {\xi_{1} ,\xi_{2} ,\xi_{3} ,\xi_{4} ,\xi_{5} ,\xi_{6} ,\xi_{7} ,\xi_{8} } \right) \in \Re$ *, it must meet the necessary conditions for the existence of an optimal control strategy. Which must satisfy the necessary conditions for the existence of an optimal control.*

*Proof* The proof is followed similarly as mentioned in [17]

## Numerical deliberation and discussion

In this study, we developed a SEI1I2I3QCR model incorporating fractional-order delay differential equations to simulate COVID-19 transmission dynamics. The inclusion of the delay parameter was crucial, as it allowed for a realistic representation of the latency period between exposure and symptom onset, which standard models cannot capture effectively. Our results demonstrated that the delay component provided a closer alignment with real-world COVID-19 data, particularly in modeling transmission lags and peak patterns observed in affected populations. Sensitivity analyses confirmed that both the delay and the fractional order parameter α\alphaα have distinct and critical roles in governing the model’s stability, with delay modeling the natural progression of infection and α\alphaα reflecting memory effects that impact system response.

To further explore practical control strategies, we applied optimal control theory to assess the impact of targeted non-pharmaceutical interventions, such as quarantine and awareness campaigns, on reducing the infected population. Numerical simulations, based on Pontryagin’s Maximum Principle, produced control profiles showing the optimal implementation of quarantine and awareness efforts over time. These simulations revealed that our control measures effectively reduced infection rates when applied early in the epidemic, particularly in the asymptomatic population. Control profiles and infection trajectory plots, included here for the first time, provide strong visual evidence of the optimal control’s effectiveness and the potential of this model to support public health decision-making.

Our findings underscore the significance of delay parameters in epidemiological models for their ability to enhance the realism and predictive power of disease transmission simulations. By combining delay differential equations with fractional-order components, the SEI1I2I3QCR model offers a novel approach to studying infectious disease dynamics and optimizing control strategies. Future studies may expand on this model to incorporate additional real-time data sources or explore applications in other infectious diseases with latency periods. This approach opens avenues for developing more sophisticated control strategies, ultimately supporting efforts to mitigate the spread of pandemics efficiently.

This section focuses mainly on the impact of the proposed model, the awareness campaigns, and the sensitivity of the basic reproduction number based on the state variables $I_{1} \left( t \right)$,$I_{2} \left( t \right)$,$I_{3} \left( t \right)$ and transmissible parameters of system (4). By implementing numerical simulations, we can illustrate our theoretical results which helps epidemiologist and stakeholders create more effective strategies for Infectious diseases that can be modeled in this compartmental transmission model.

To ensure the reliability and reproducibility of our proposed SEI1I2I3QCR model integrating fractional-order delay differential equations, the methodology section provides a detailed account of all modeling assumptions, parameter definitions, numerical implementation, and control strategies.

### Optimal solution

The optimal solution derived from the SEI1I2I3QCR model underscores the critical role of strategically implemented control measures in managing the spread of COVID-19. Our analysis reveals that the optimal combination of quarantine and awareness campaign strategies significantly reduces the effective reproduction number (R0), facilitating a decline in new infections over time. The results indicate that initiating awareness campaigns at the onset of an outbreak not only accelerates the identification and isolation of symptomatic individuals but also fosters a culture of compliance among the population, leading to a higher proportion of individuals seeking testing and self-isolating when symptomatic.

Furthermore, the model illustrates that varying the intensity of control measures throughout the epidemic can yield substantial benefits. Specifically, aggressive quarantine measures during peak transmission periods are shown to be highly effective in curtailing the number of pre-symptomatic and mildly symptomatic individuals who would otherwise contribute to further spread. The simulations suggest that a phased approach- whereby awareness campaigns are ramped up during initial outbreaks followed by tailored quarantine responses as infection rates change produces the most favorable outcomes in terms of both health and economic costs.

The optimal solution also highlights the importance of flexibility in intervention strategies; as the epidemic evolves, the model advocates for continuous assessment and adjustment of control measures based on real-time data. This adaptability is vital in maintaining low transmission rates and preventing the resurgence of infections. Overall, the findings from the optimal solution analysis provide valuable insights for policymakers, emphasizing that effective control of infectious diseases hinges not only on immediate interventions but also on the foresight to adapt strategies dynamically in response to changing epidemiological landscapes.

In this subsection, we mainly focus on the impact of the controlling parameter, the isolation rate of the infectious individuals, and time delay on the spread of the disease. Firstly we take set 1 of the parameters and initial conditions which define in system (1) and tabulated in Table 1, the compartments elaborate the existence of populations in susceptible and infectious compartments, which slowly transmit into the other compartments over time. Furthermore, simulations of all compartments with and without controlling strategies are investigated and represented using the software Mathematica 12.1, which includes built-in Runge–Kutta programming.

**Fig. 3:**
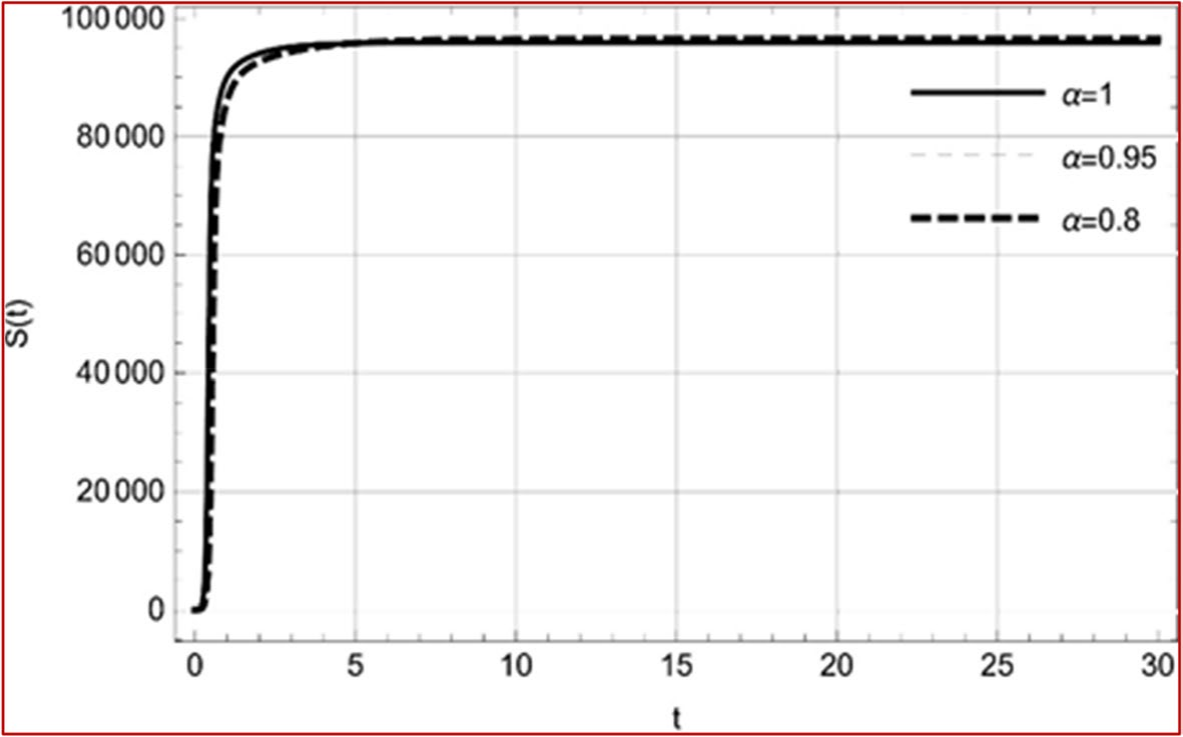
Memory profile of $S\left( t \right) \in \Gamma_{1}$ of system (4), for parameters describe in set 1 of Table 1 at $\tau_{1} = \tau_{2} = 0$ and $u = { 0}{\text{.8357}}$ for $\alpha = 0.8,\;0.95,\;1$ and $t \in \left[ {0,30} \right]$

**Fig. 4:**
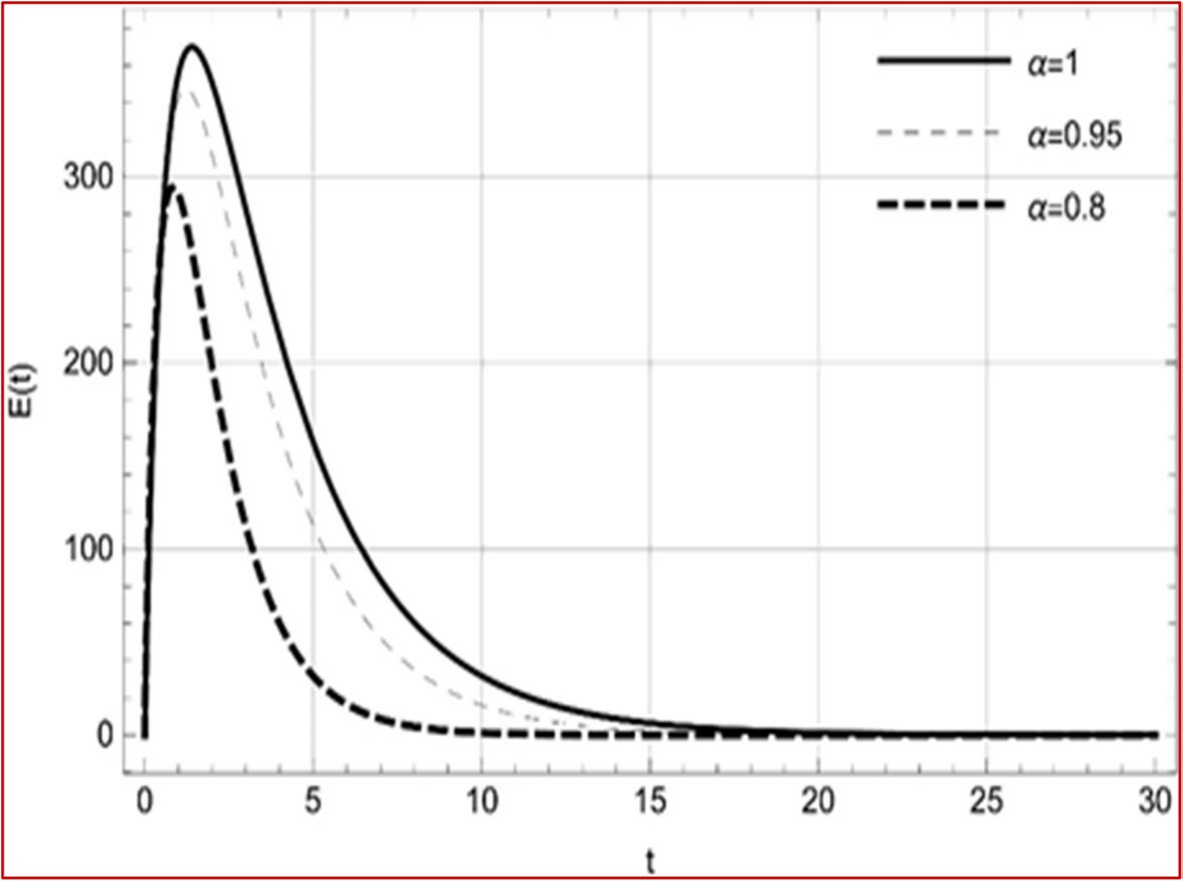
Memory profile of $E\left( t \right) \in \Gamma_{1}$ of system (4), for parameters describe in set 1 of Table 1 at $\tau_{1} = \tau_{2} = 0$ and $u = { 0}{\text{.8357}}$ for $\alpha = 0.8,\;0.95,\;1$ and $t \in \left[ {0,30} \right]$

**Fig. 5:**
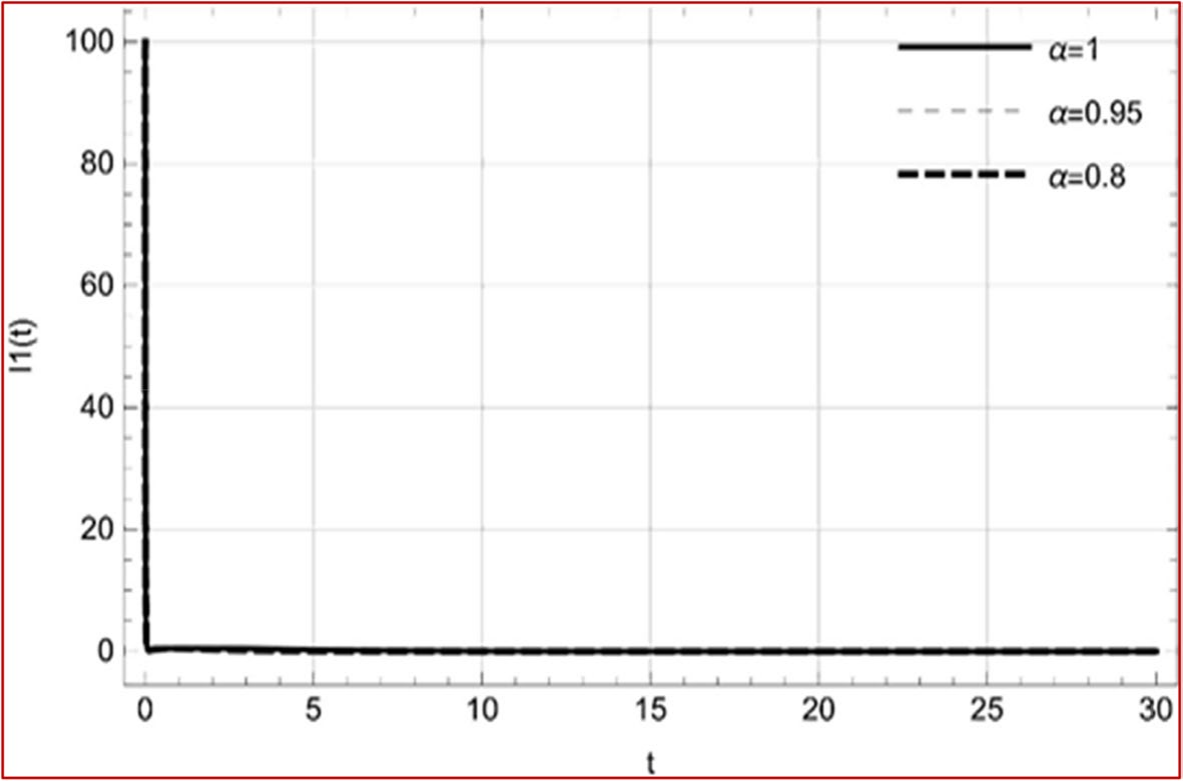
Memory profile of $I_{1} \left( t \right) \in \Gamma_{1}$ of system (4), for parameters describe in set 1 of Table 1 at $\tau_{1} = \tau_{2} = 0$ and $u = { 0}{\text{.8357}}$ for $\alpha = 0.8,\;0.95,\;1$ and $t \in \left[ {0,30} \right]$

**Fig. 6:**
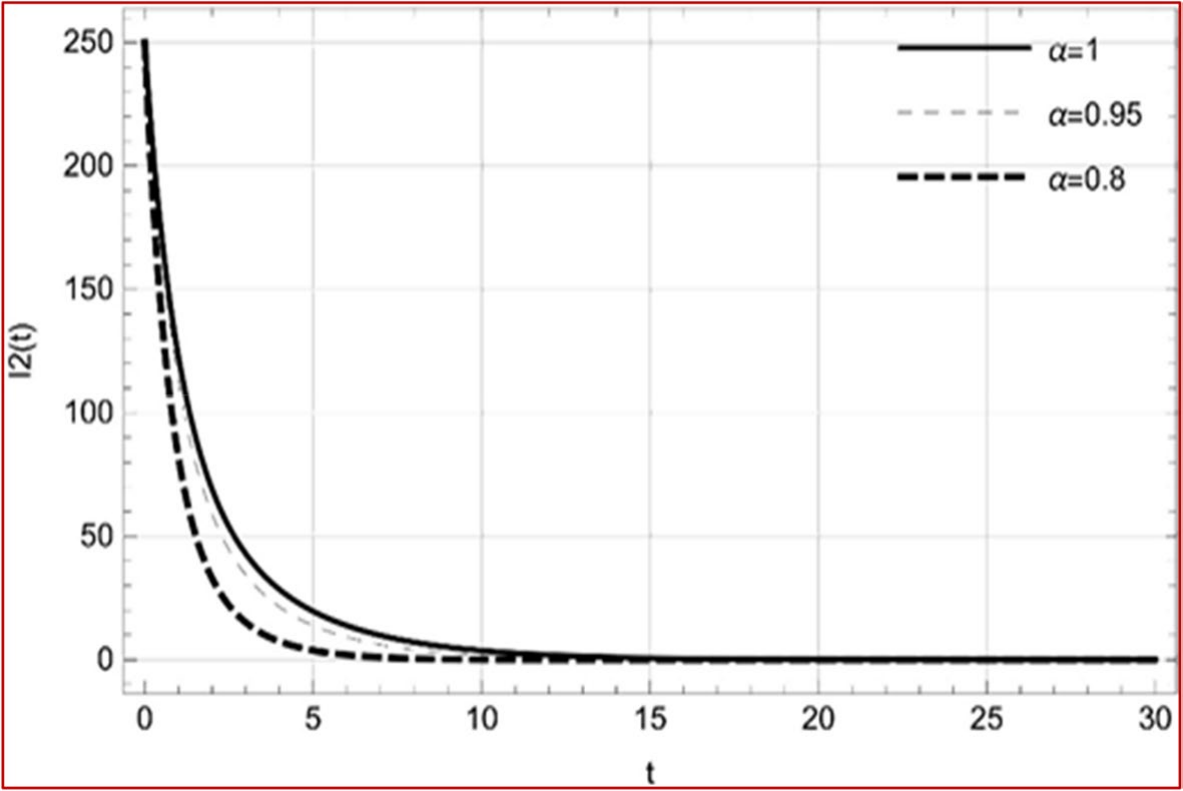
Memory profile of $I_{2} \left( t \right) \in \Gamma_{1}$ of system (4), for parameters describe in set 1 of Table 1 at $\tau_{1} = \tau_{2} = 0$ and $u = { 0}{\text{.8357}}$ for $\alpha = 0.8,\;0.95,\;1$ and $t \in \left[ {0,30} \right]$

**Fig. 7:**
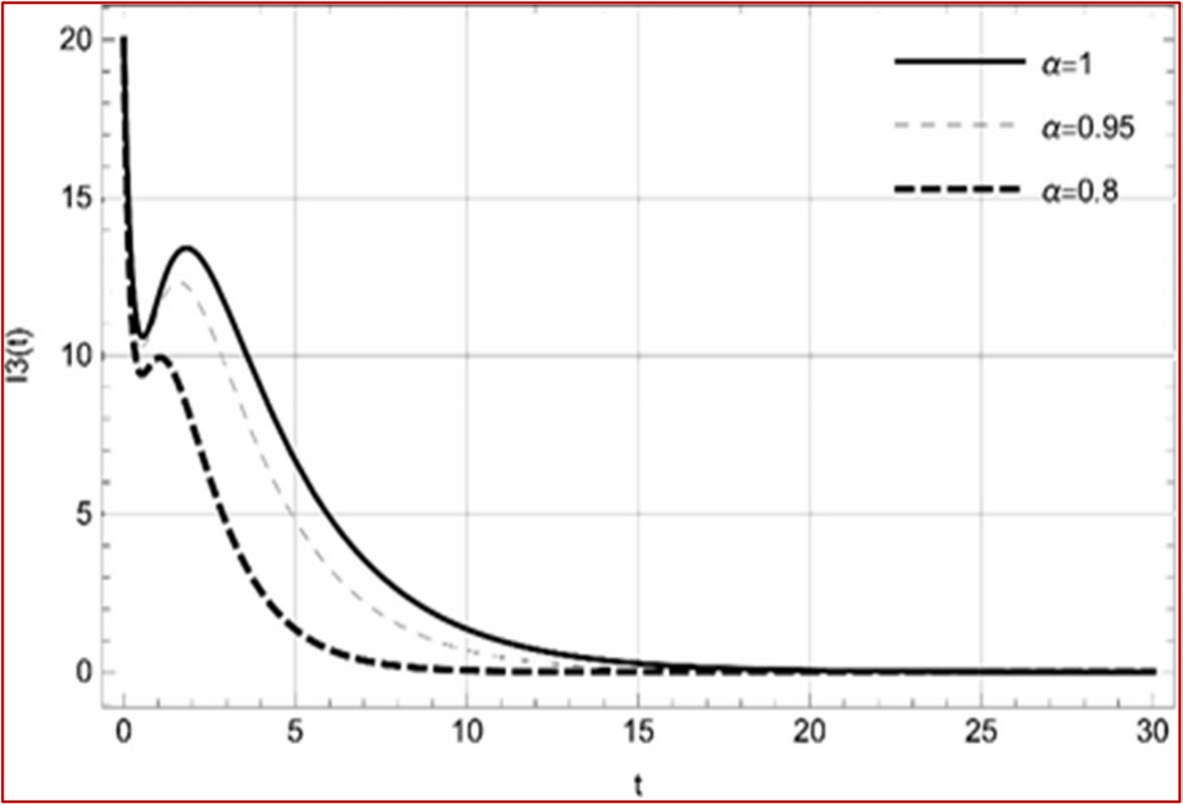
Memory profile of $I_{3} \left( t \right) \in \Gamma_{1}$ of system (4), for parameters describe in set 1 of Table 1 at $\tau_{1} = \tau_{2} = 0$ and $u = { 0}{\text{.8357}}$ for $\alpha = 0.8,\;0.95,\;1$ and $t \in \left[ {0,30} \right]$

**Fig. 8:**
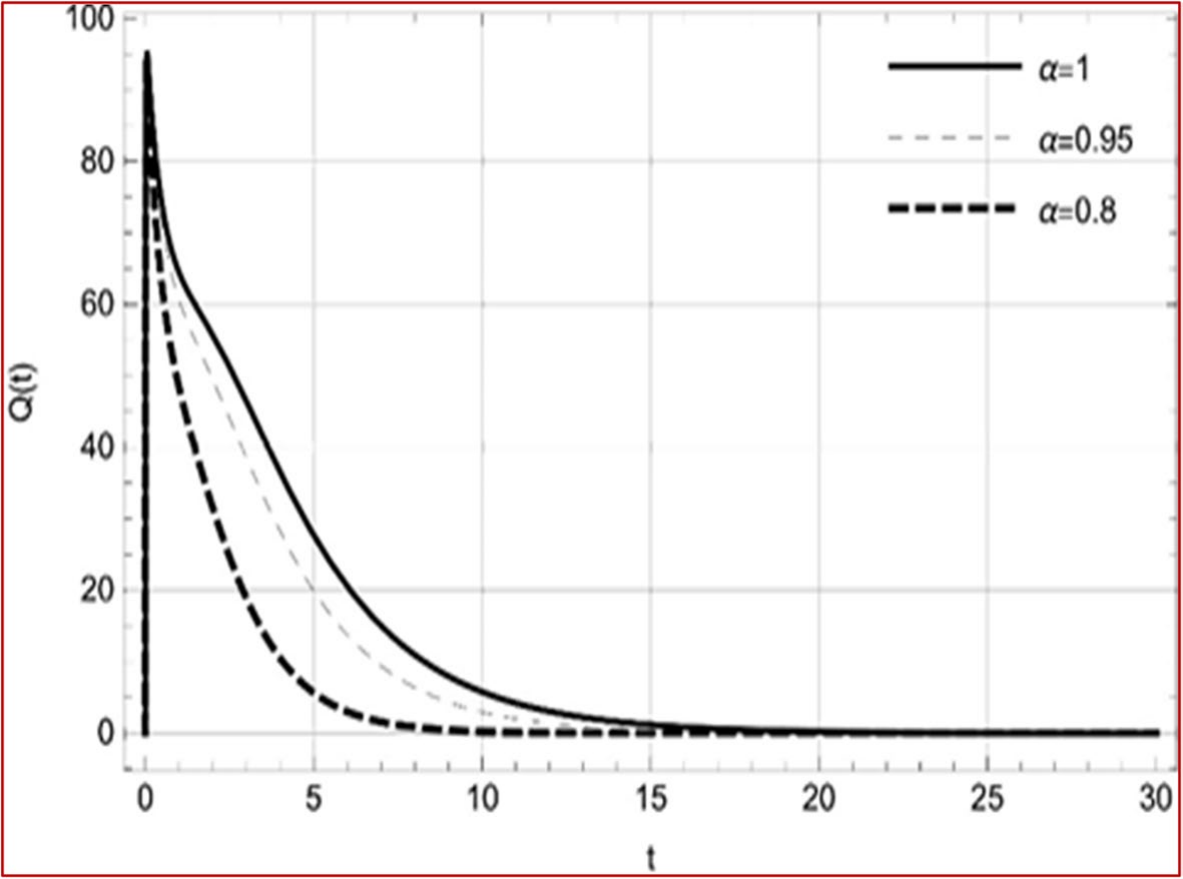
Memory profile of $Q\left( t \right) \in \Gamma_{1}$ of system (4), for parameters describe in set 1 of Table 1 at $\tau_{1} = \tau_{2} = 0$ and $u = { 0}{\text{.8357}}$ for $\alpha = 0.8,\;0.95,\;1$ and $t \in \left[ {0,30} \right]$

**Fig. 9:**
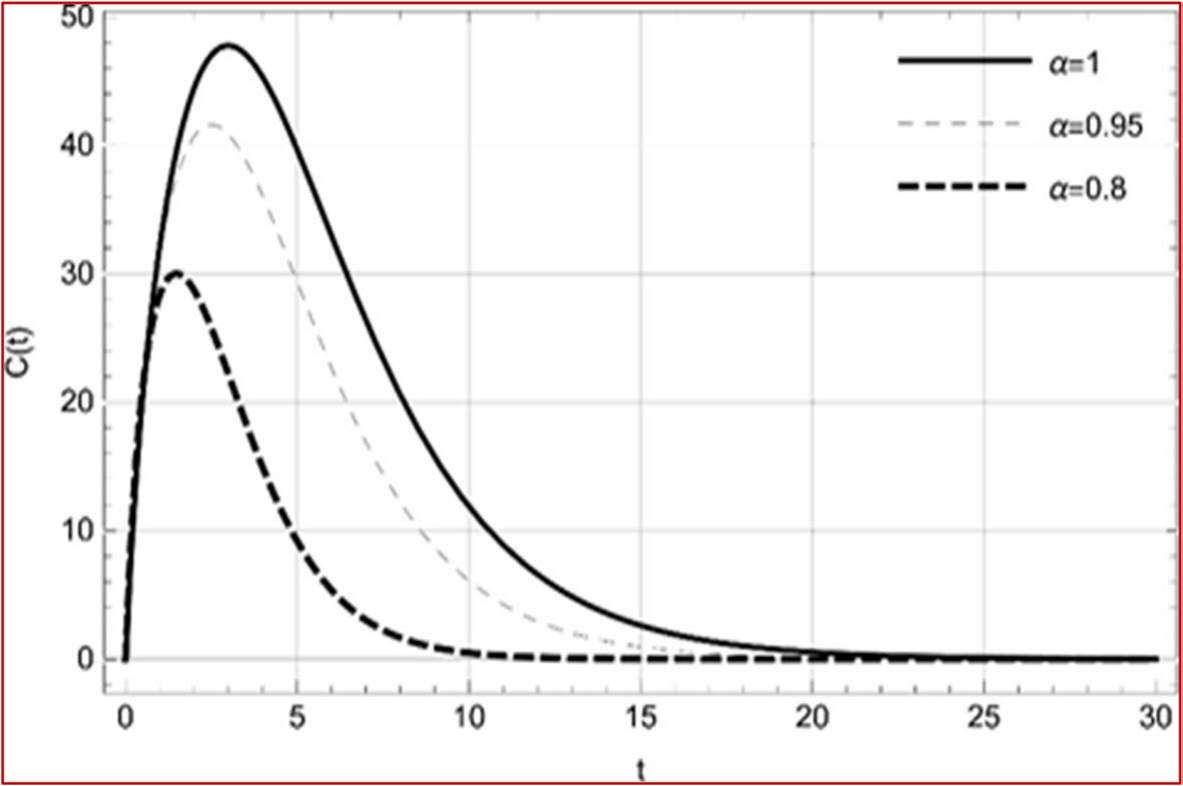
Memory profile of $C\left( t \right) \in \Gamma_{1}$ of system (4), for parameters describe in set 1 of Table 1 at $\tau_{1} = \tau_{2} = 0$ and $u = { 0}{\text{.47379 }}$ for $\alpha = 0.8,\;0.95,\;1$ and $t \in \left[ {0,30} \right]$

**Fig. 10:**
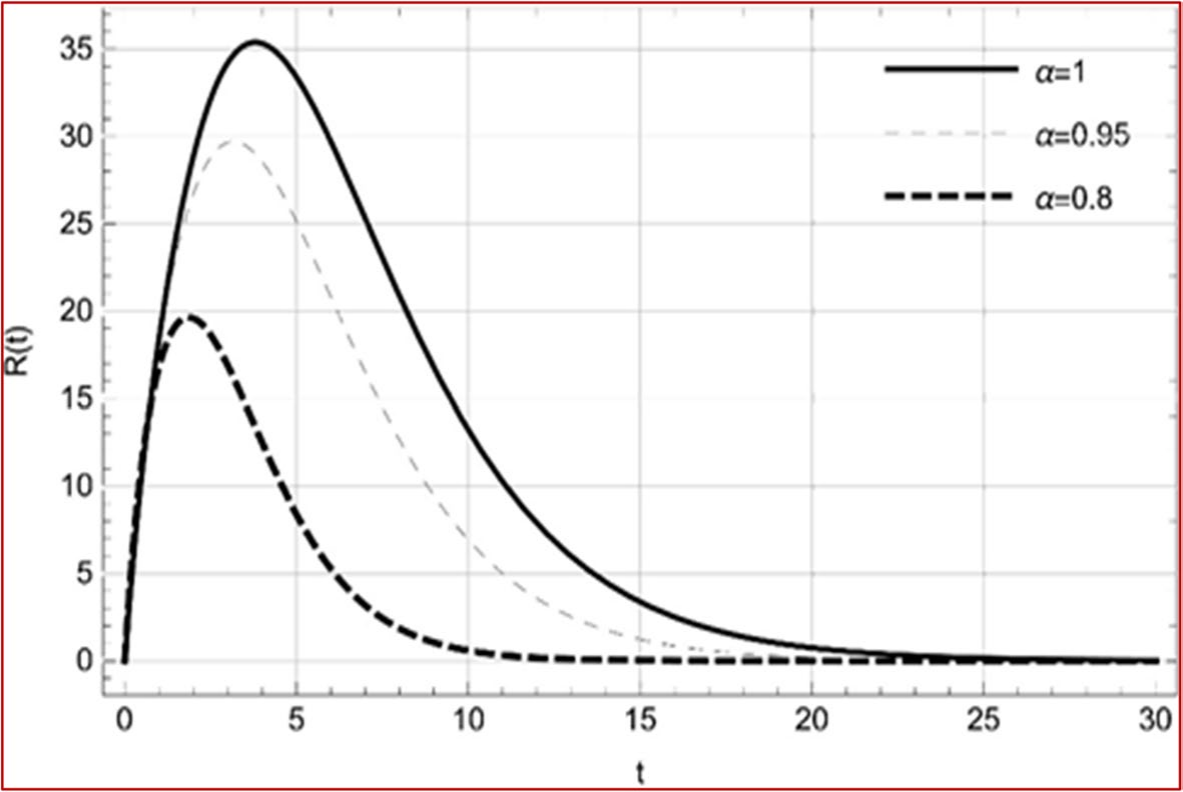
Memory profile of $R\left( t \right) \in \Gamma_{1}$ of system (4), for parameters describe in set 1 of Table 1 at $\tau_{1} = \tau_{2} = 0$ and $u = { 0}{\text{.8357}}$ for $\alpha = 0.8,\;0.95,\;1$ and $t \in \left[ {0,30} \right]$

**Fig. 11:**
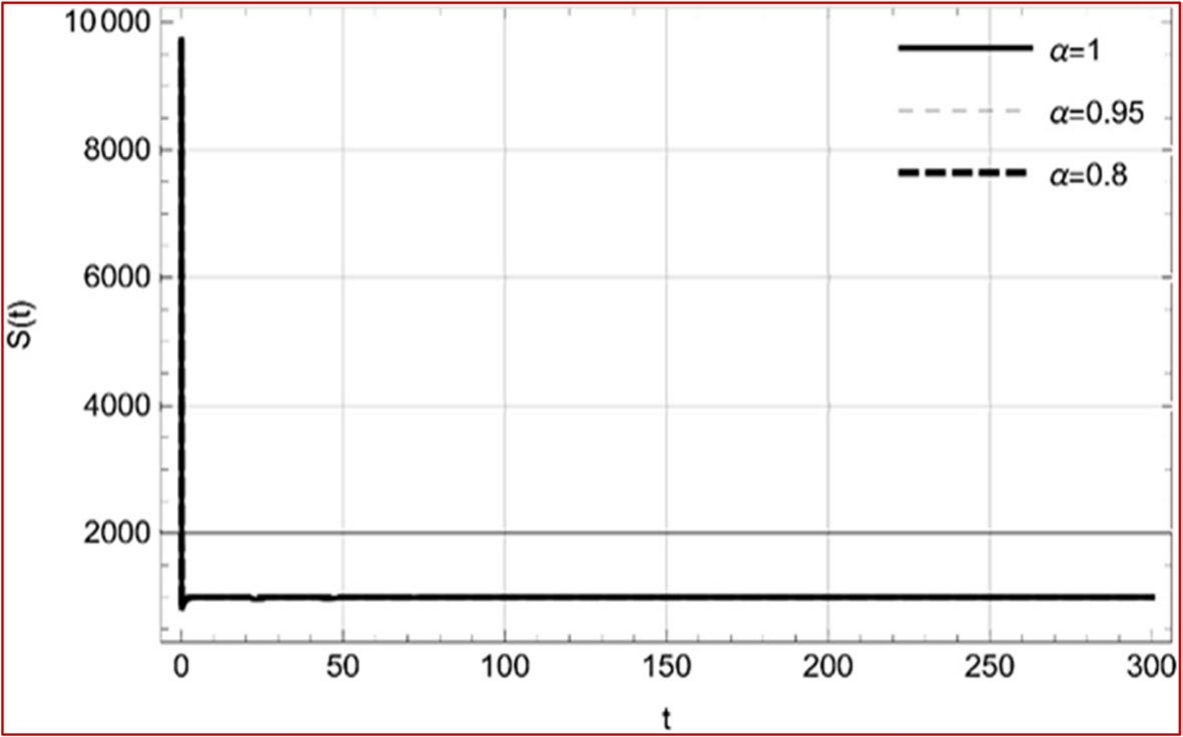
Memory profile of $S\left( t \right) \in \Gamma_{1}$ of system (4), for parameters, described in set 1 of Table 1 at $\tau_{1} = 1.375,\tau_{2} = 2.35$ and $u = { 0}{\text{.8357}}$ for $\alpha = 0.8,\;0.95,\;1$ and $t \in \left[ {0,30} \right]$

**Fig. 12:**
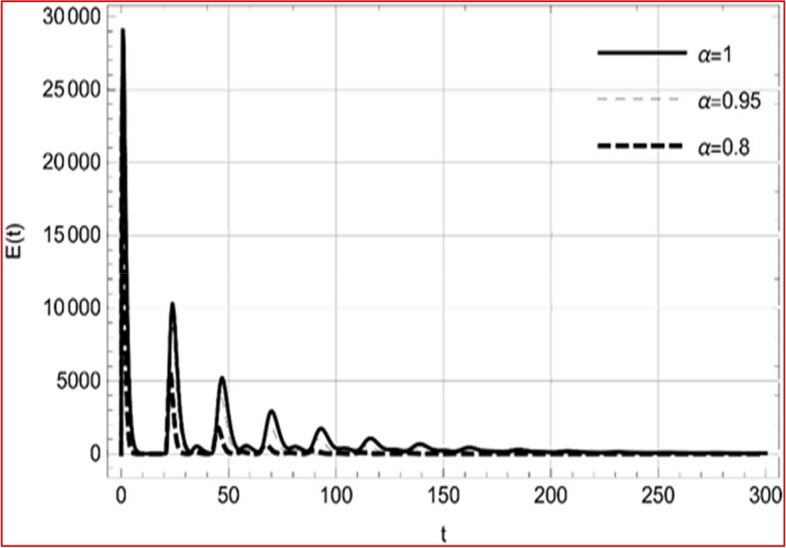
Memory profile of $E\left( t \right) \in \Gamma_{1}$ of system (4), for parameters, described in set 1 of Table 1 at $u = { 0}{\text{.8357}}$ for $\alpha = 0.8,\;0.95,\;1$ and $t \in \left[ {0,300} \right]$

**Fig. 13:**
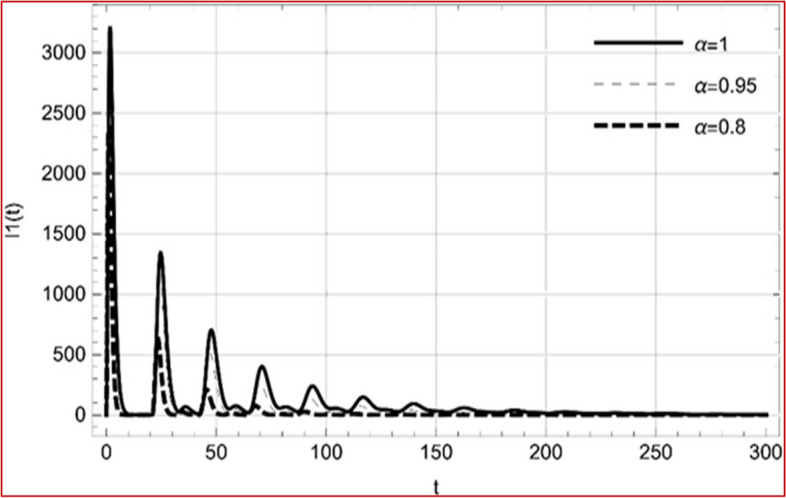
Memory profile of $I_{1} \left( t \right) \in \Gamma_{1}$ of system (4), for parameters, described in set 1 of Table 1 at $\tau_{1} = 1.375,\tau_{2} = 2.35$ and $u = { 0}{\text{.8357}}$ for $\alpha = 0.8,\;0.95,\;1$ and $t \in \left[ {0,300} \right]$

**Fig. 14:**
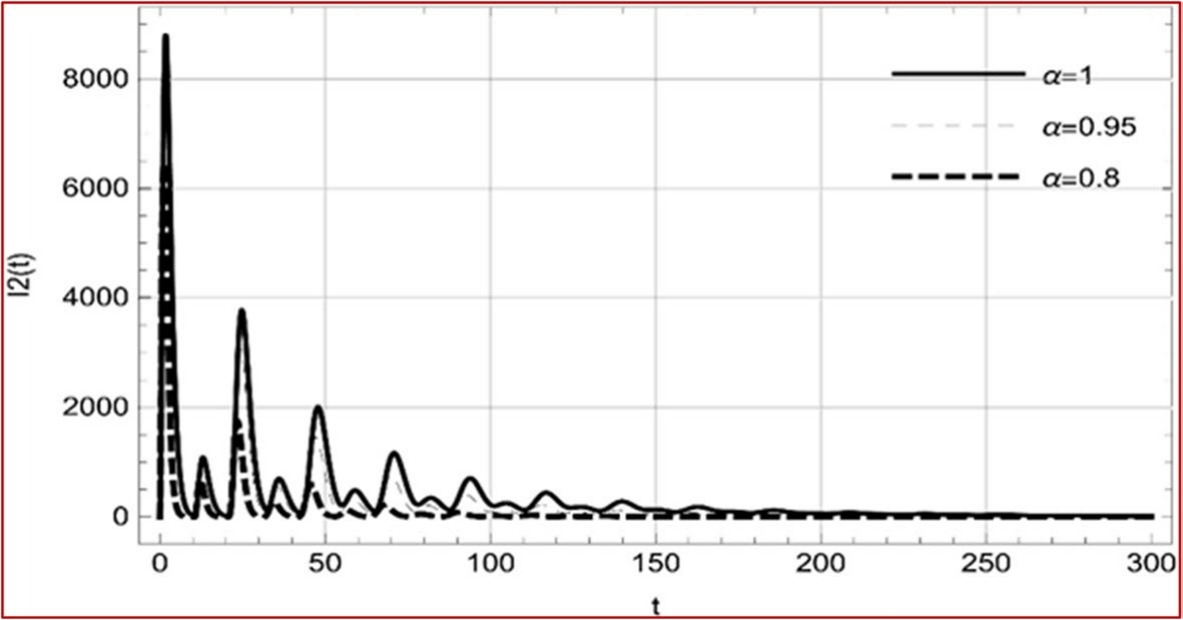
Memory profile of $I_{2} \left( t \right) \in \Gamma_{1}$ of system (4), for parameters, described in set 1 of Table 1 at $\tau_{1} = 1.375,\tau_{2} = 2.35$ and $u = { 0}{\text{.8357}}$ for $\alpha = 0.8,\;0.95,\;1$ and $t \in \left[ {0,300} \right]$

**Fig. 15:**
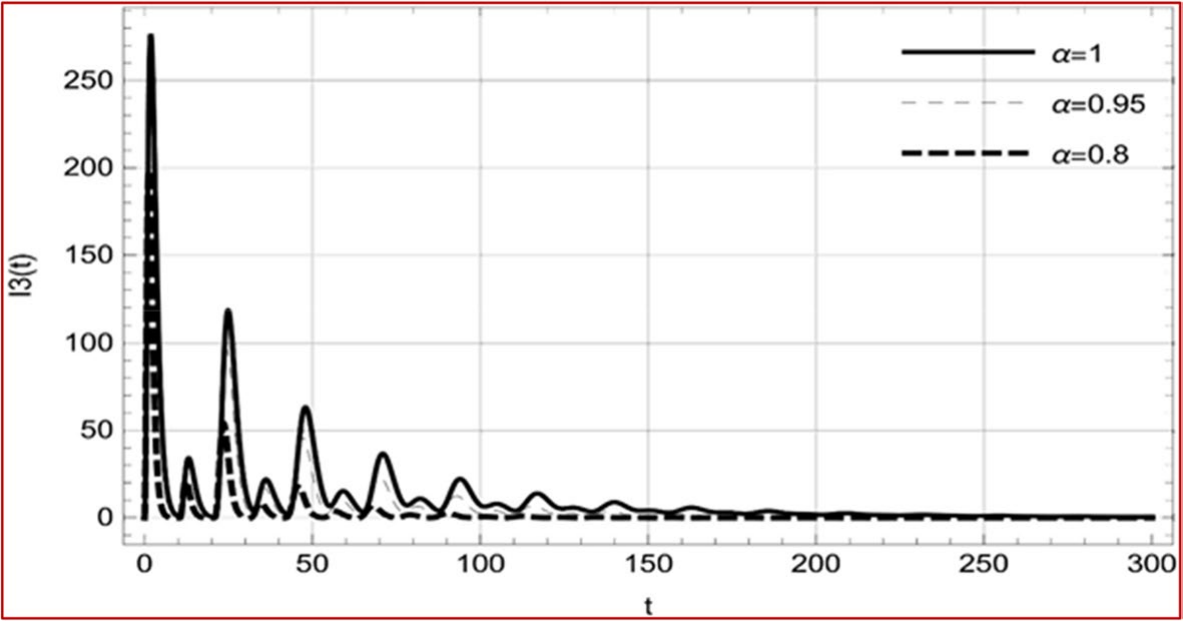
Memory profile of $I_{3} \left( t \right) \in \Gamma_{1}$ of system (4), for parameters, described in set 1 of Table 1 at $\tau_{1} = 1.375,\tau_{2} = 2.35$ and $u = { 0}{\text{.8357}}$ for $\alpha = 0.8,\;0.95,\;1$ and $t \in \left[ {0,300} \right]$

**Fig. 16:**
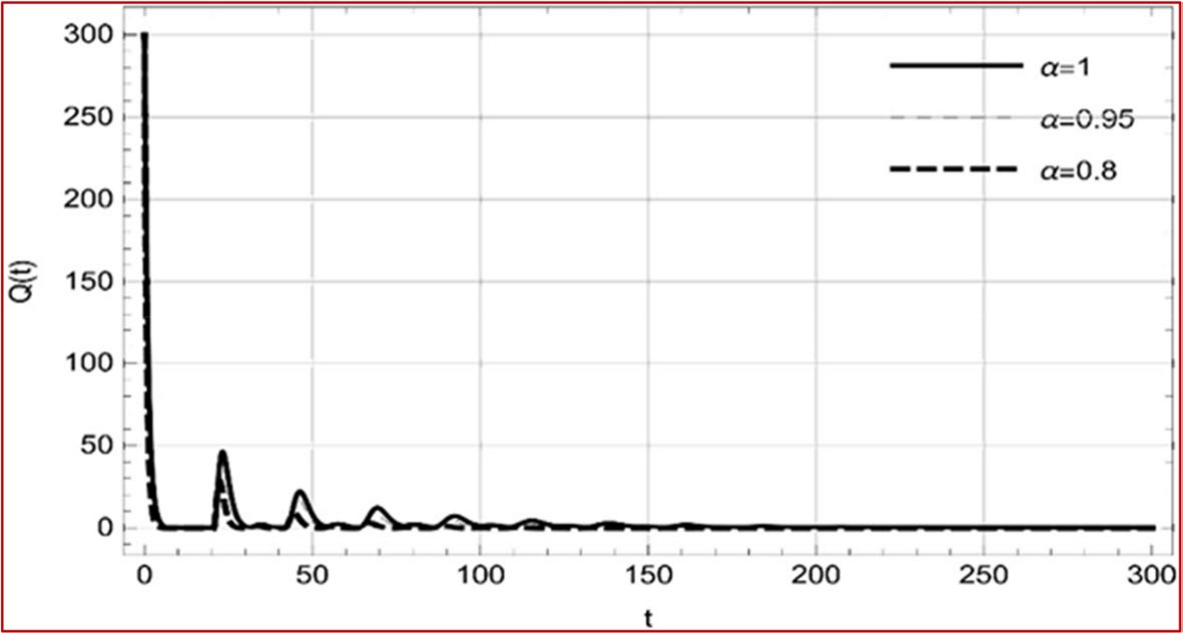
Memory profile of $Q\left( t \right) \in \Gamma_{1}$ of system (4), for parameters, described in set 1 of Table 1 at $\tau_{1} = 1.375,\tau_{2} = 2.35$ and $u = { 0}{\text{.8357}}$ for $\alpha = 0.8,\;0.95,\;1$ and $t \in \left[ {0,300} \right]$

**Fig. 17:**
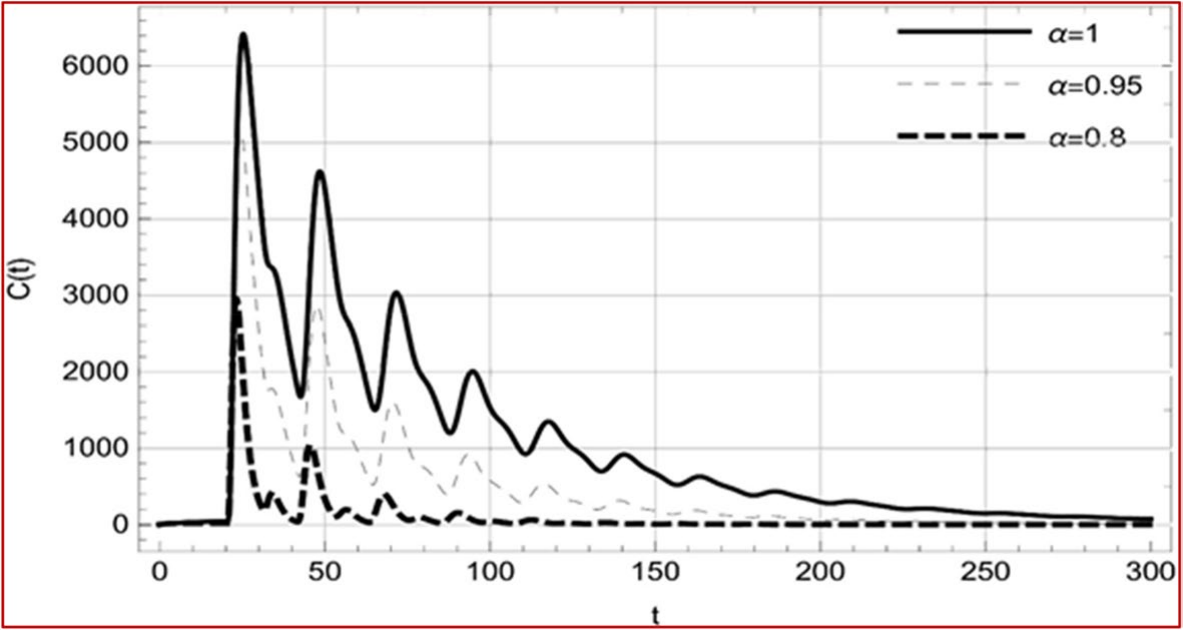
Memory profile of $C\left( t \right) \in \Gamma_{1}$ of system (4), for parameters, described in set 1 of Table 1 at $\tau_{1} = 1.375,\tau_{2} = 2.35$ and $u = { 0}{\text{.8357}}$ for $\alpha = 0.8,\;0.95,\;1$ and $t \in \left[ {0,300} \right]$

**Fig. 18:**
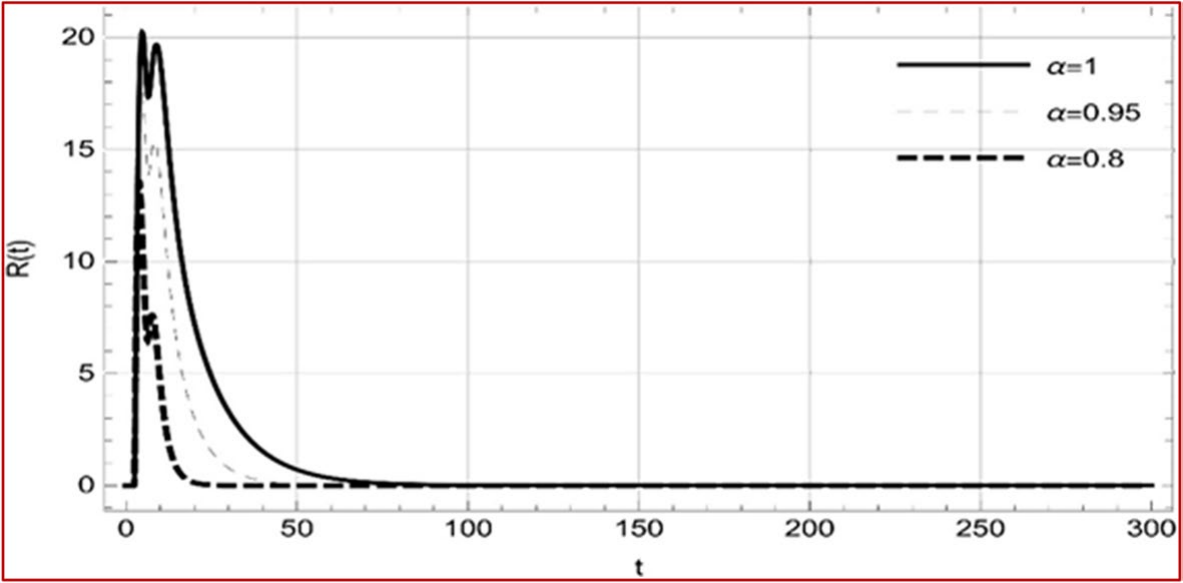
Memory profile of $R\left( t \right) \in \Gamma_{1}$ of system (4), for parameters, described in set 1 of Table 1 at $\tau_{1} = 1.375,\tau_{2} = 2.35$ and $u = { 0}{\text{.8357}}$ for $\alpha = 0.8,\;0.95,\;1$ and $t \in \left[ {0,300} \right]$

With set 1 of Table 1 at different fractional orders, the reproduction number and constant solutions are computed, indicating that $S\left( t \right)$ is the only location for the whole population with range of 298.537 to 299.712 thousand, satisfying the disease-free equilibrium point and stability results of Theorem 4. For this purpose, parameter $u$ and transmission rates $\beta_{1} ,\beta_{2}$ and $\beta_{3} ,$ played a significant role in finding these values, which showed that gradual reductions in transmission rates lead to smaller reproduction numbers. Figures 3, 4, 5, 6, 7, 8, 9 and 10 illustrate the dynamical effects of the solution of system (4) showing that the disease has disappeared from the population when controlling parameter $u = 0.8573$ and existence of disease-free equilibrium in the absence of time delay. Beside this the existence of time delay to goes out the stability of disease free state are represent in Figs. 11, 12, 13, 14, 15, 16, 17 and 18 where we can easily see that the disease die out after the time lag of $\tau_{1} = 1.375$ and $\tau_{2} = 0.124$ for $t \in \left[ {0,300} \right]$. Therefore the number of asymptomatic cases gradually decreases when infected individuals become aware of the mortality and transmission of disease, and voluntarily enter the quarantine. Hence disease free equilibrium in each compartment of system (4) is achieved over time.

**Fig. 19:**
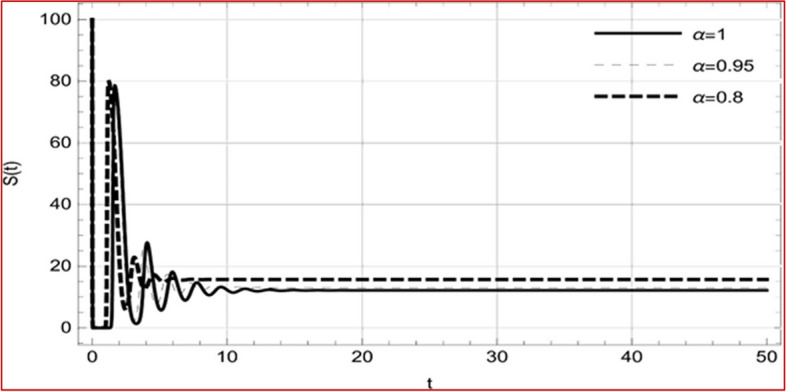
Memory profile of $S\left( t \right) \in \Gamma_{2}$ of system (4), for parameters describe in set 1 of Table 1 at $\tau_{1} = \tau_{2} = 0$ and $u = { 0}$ for $\alpha = 0.8,\;0.95,\;1$ and $t \in \left[ {0,50} \right]$

**Fig. 20:**
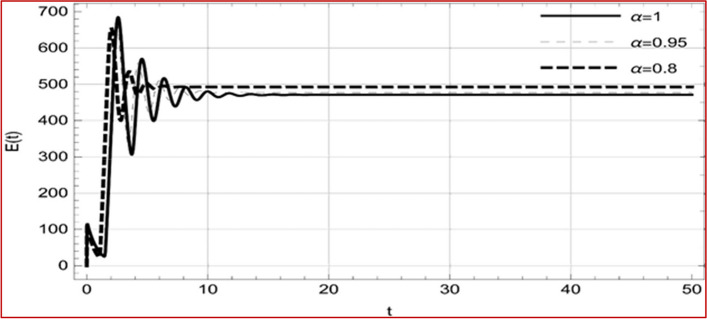
Memory profile of $E\left( t \right) \in \Gamma_{2}$ of system (4), for parameters, describe in set 1 of Table 1 at $\tau_{1}=\tau_{2}=0$ and $u = { 0}$ for $\alpha = 0.8,\;0.95,\;1$ and $t \in \left[ {0,50} \right]$

**Fig. 21:**
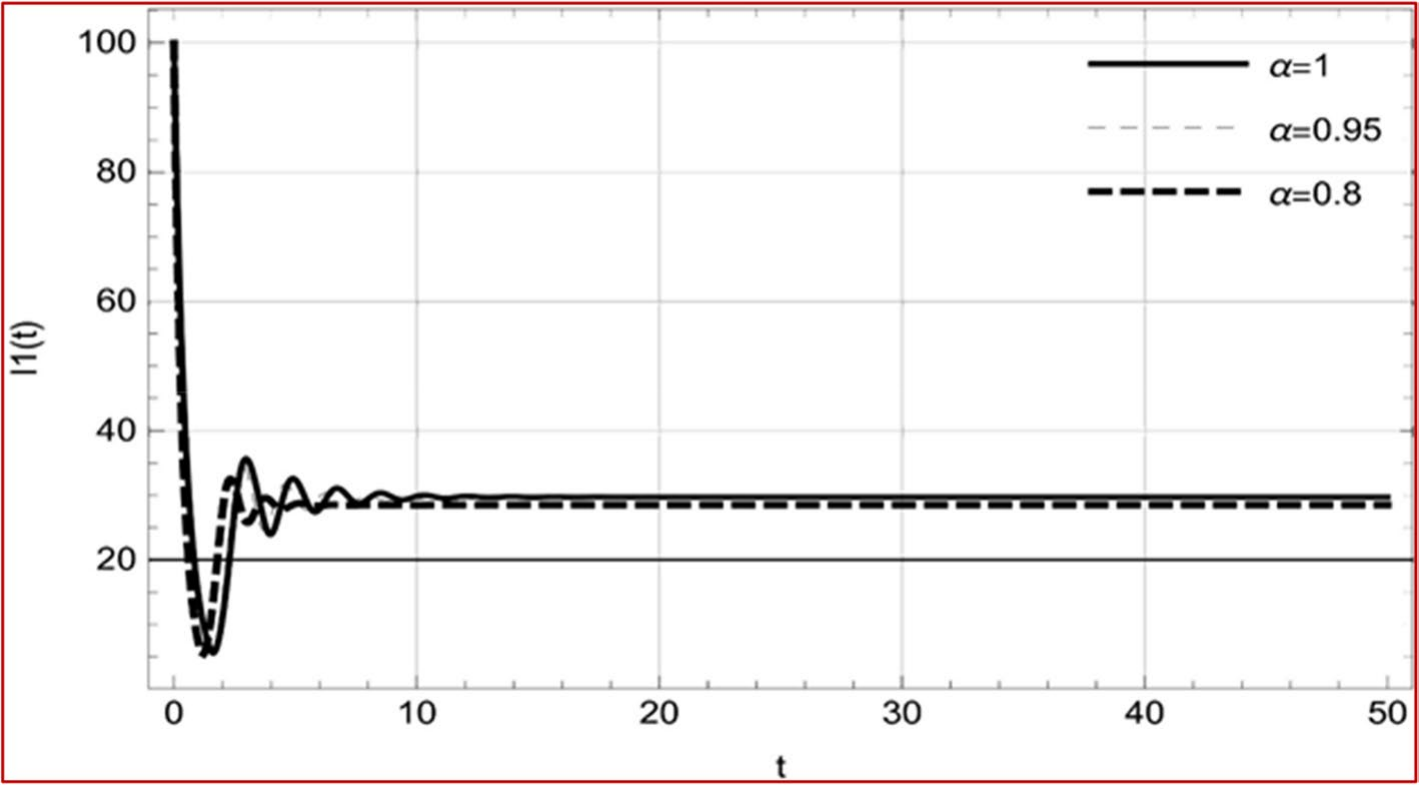
Memory profile of $I_{1} \left( t \right) \in \Gamma_{2}$ of system (4), for parameters describe in set 1 of Table 1 at $\tau_{1} = \tau_{2} = 0$ and $u = { 0}$ for $\alpha = 0.8,\;0.95,\;1$ and $t \in \left[ {0,50} \right]$

**Fig. 22:**
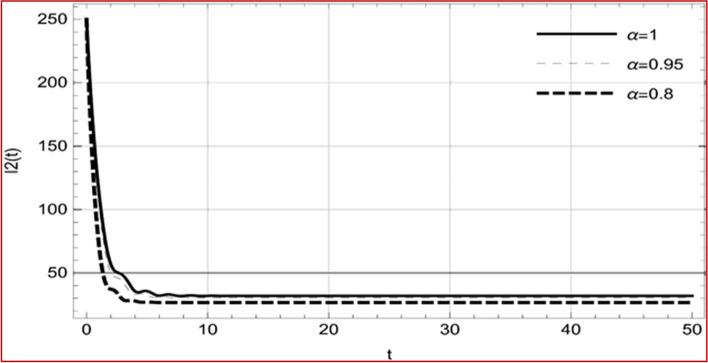
Memory profile of $I_{2} \left( t \right) \in \Gamma_{2}$ of system (4), for parameters describe in set 1 of Table 1 at $\tau_{1} = \tau_{2} = 0$ and $u = { 0}$ for $\alpha = 0.8,\;0.95,\;1$ and $t \in \left[ {0,50} \right]$

**Fig. 23:**
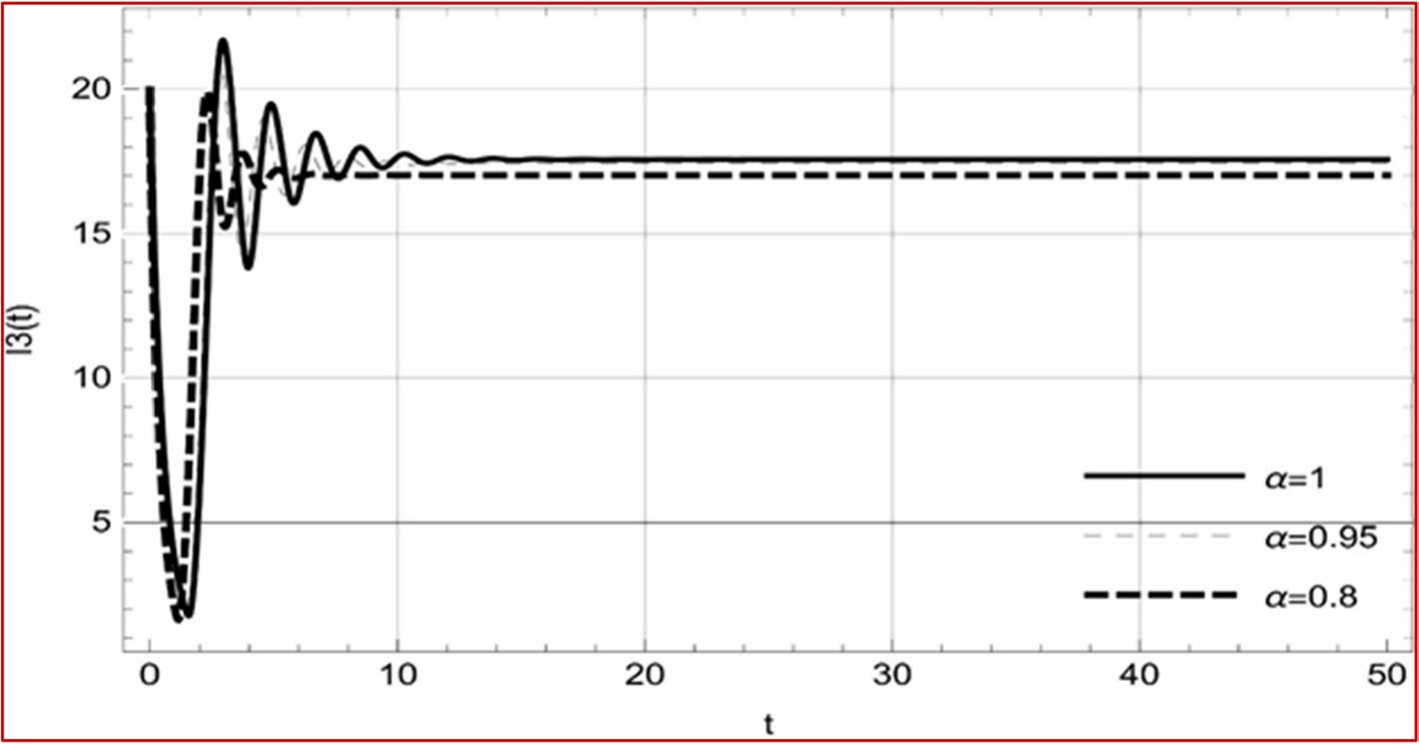
Memory profile of $I_{3} \left( t \right) \in \Gamma_{2}$ of system (4), for parameters describe in set 1 of Table 1 at $\tau_{1} = \tau_{2} = 0$ and $u = { 0}$ for $\alpha = 0.8,\;0.95,\;1$ and $t \in \left[ {0,50} \right]$

**Fig. 24:**
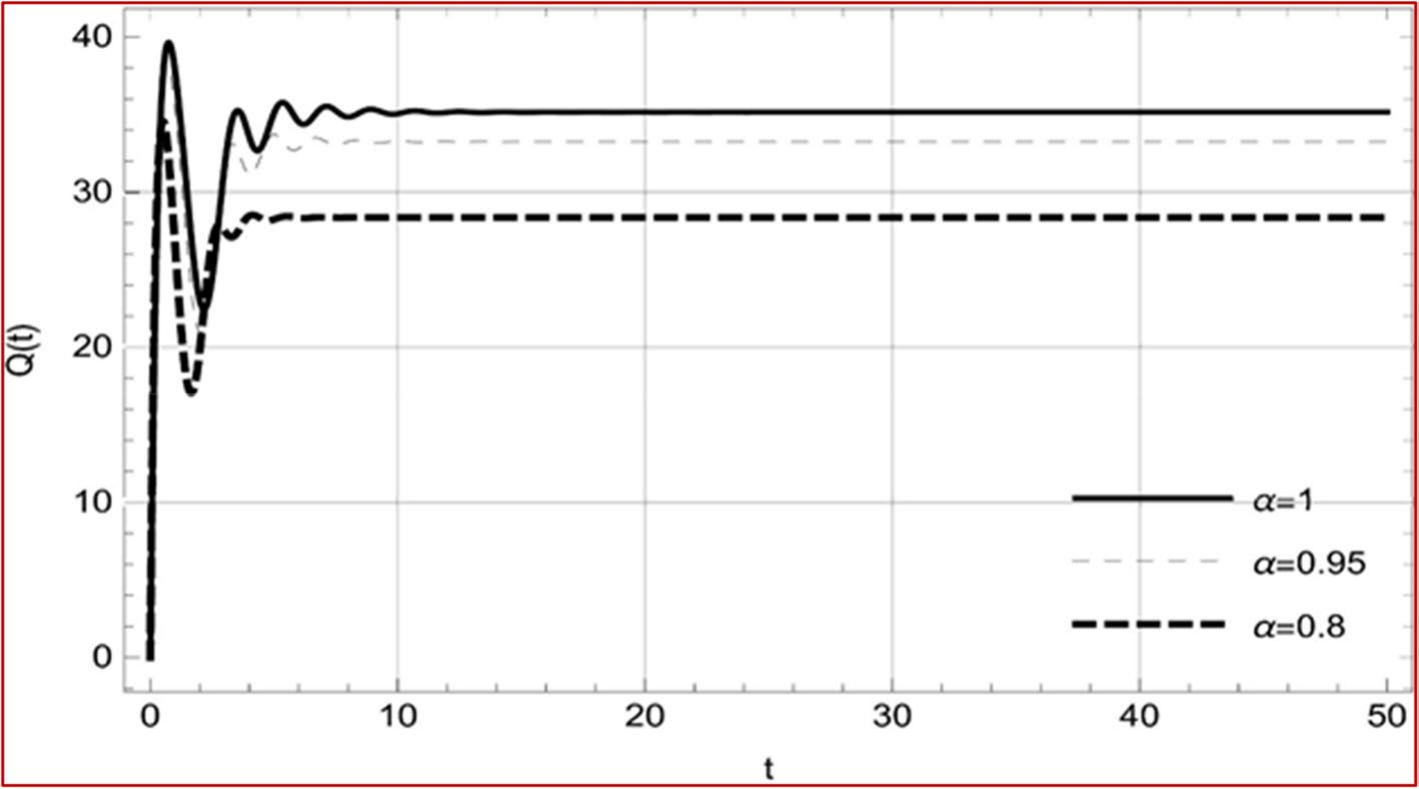
Memory profile of $Q\left( t \right) \in \Gamma_{2}$ of system (4), for parameters describe in set 1 of Table 1 at $\tau_{1} = \tau_{2} = 0$ and $u = { 0}$ for $\alpha = 0.8,\;0.95,\;1$ and $t \in \left[ {0,50} \right]$

**Fig. 25:**
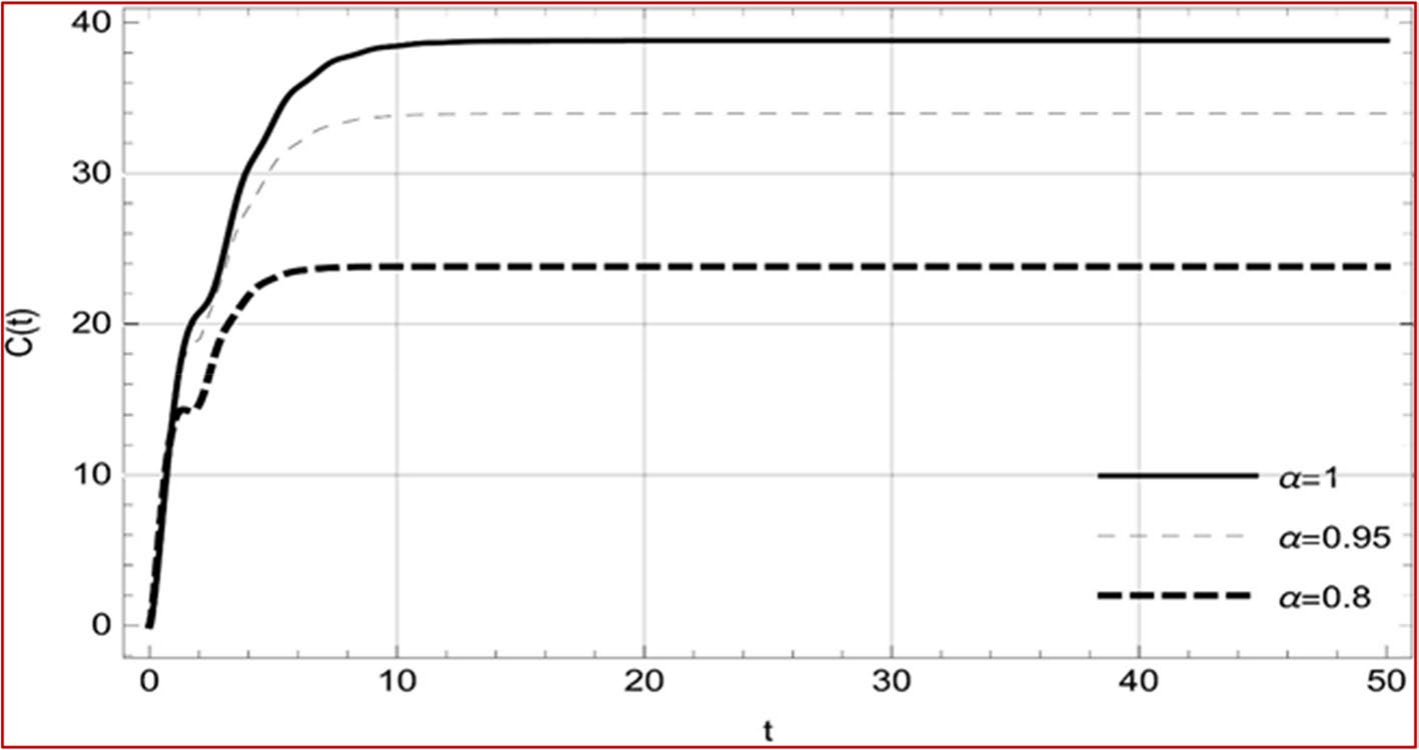
Memory profile of $C\left( t \right) \in \Gamma_{2}$ of system (4), for parameters describe in set 1 of Table 1 at $\tau_{1} = \tau_{2} = 0$ and $u = { 0}$ for $\alpha = 0.8,\;0.95,\;1$ and $t \in \left[ {0,50} \right]$

**Fig. 26:**
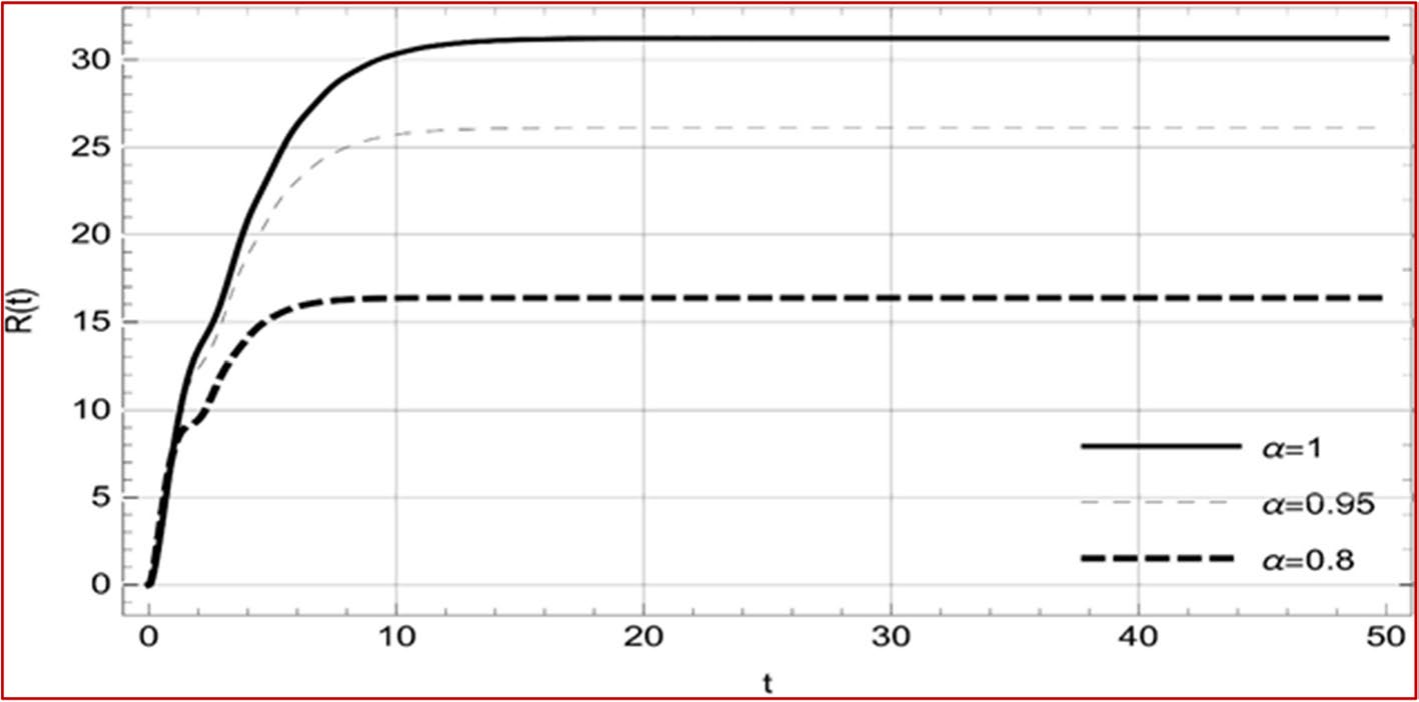
Memory profile of $R\left( t \right) \in \Gamma_{2}$ of system (4), for parameters describe in set 1 of Table 1 at $\tau_{1} = \tau_{2} = 0$ and $u = { 0}$ for $\alpha = 0.8,\;0.95,\;1$ and $t \in \left[ {0,50} \right]$

**Fig. 27:**
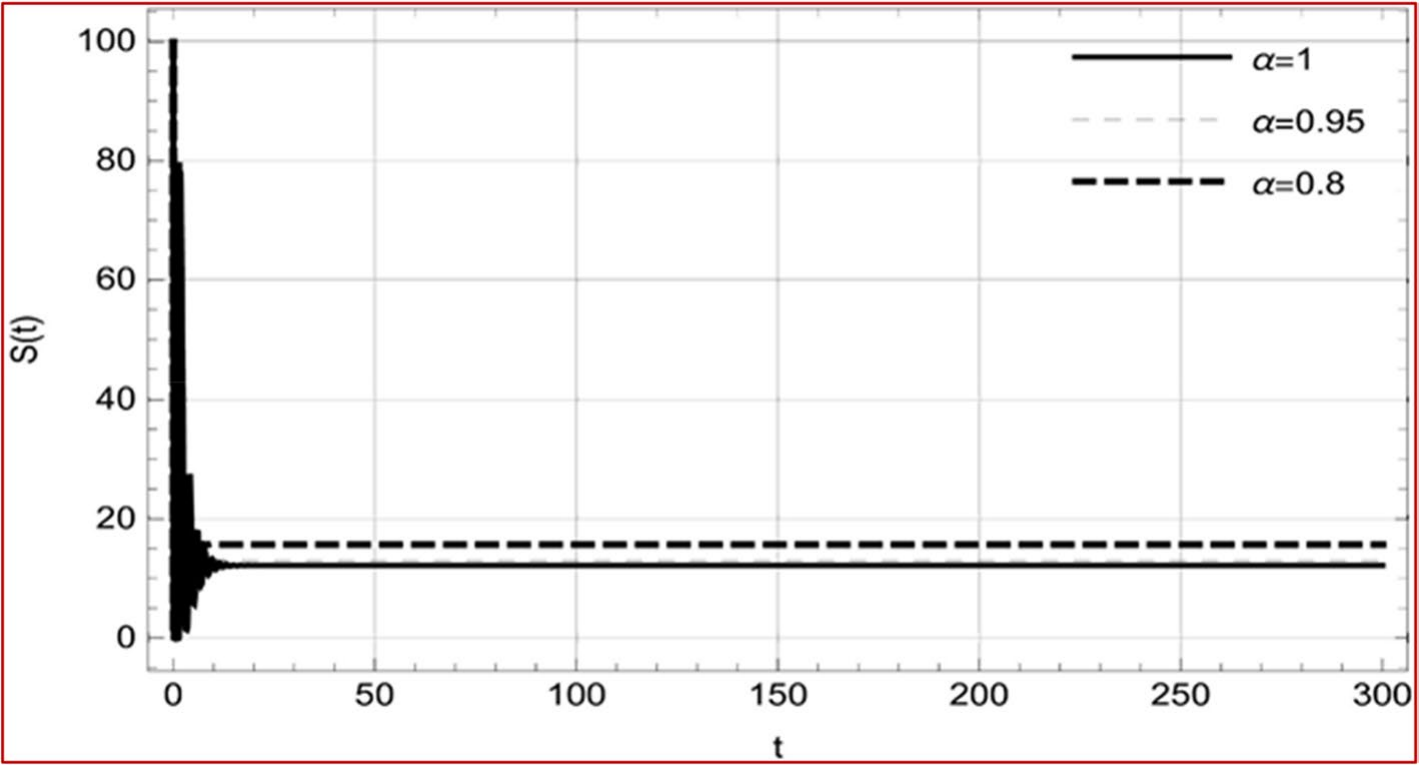
Memory profile of $S\left( t \right) \in \Gamma_{2}$ of system (4), for parameters describe in set 1 of Table 1 at $\tau_{1} = 1.375,\tau_{2} = 2.5$ and $u = { 0}$ for $\alpha = 0.8,\;0.95,\;1$ and $t \in \left[ {0,300} \right]$

**Fig. 28:**
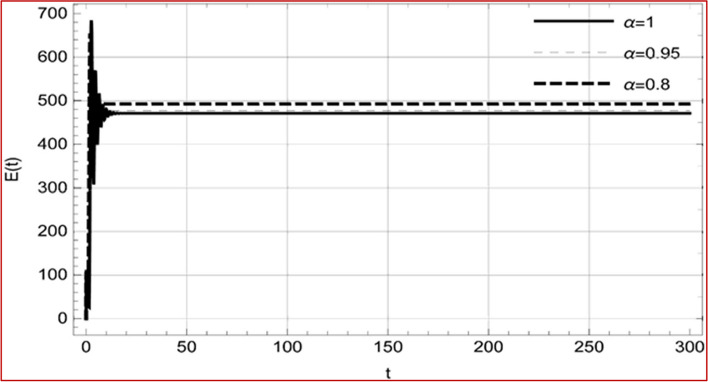
Memory profile of $E\left( t \right) \in \Gamma_{2}$ of system (4), for parameters describe in set 1 of Table 1 at $\tau_{1} = 1.375,\tau_{2} = 2.5$ and $u = { 0}$ for $\alpha = 0.8,\;0.95,\;1$ and $t \in \left[ {0,300} \right]$

**Fig. 29:**
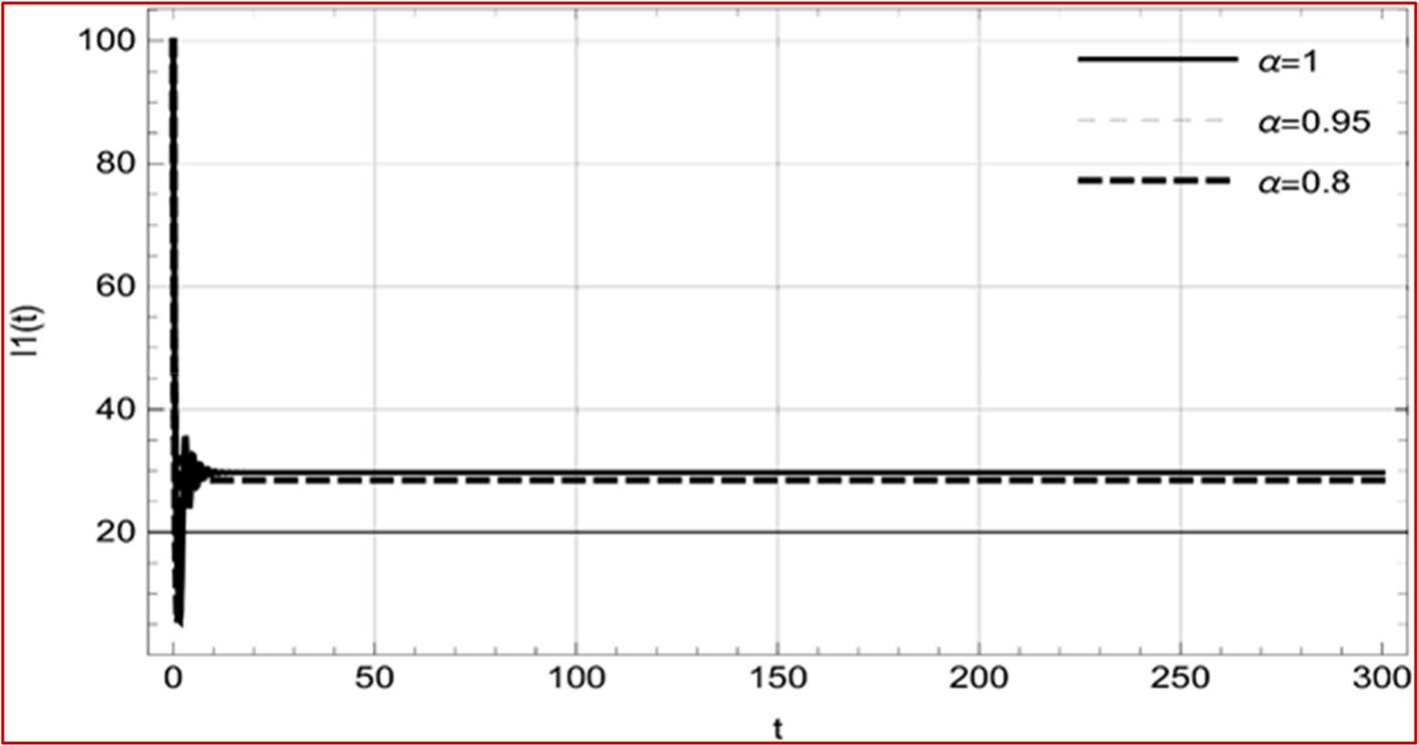
Memory profile of $I_{1} \left( t \right) \in \Gamma_{2}$ of system (4), for parameters describe in set 1 of Table 1 at $\tau_{1} = 1.375,\tau_{2} = 2.5$ and $u = { 0}$ for $\alpha = 0.8,\;0.95,\;1$ and $t \in \left[ {0,300} \right]$

**Fig. 30:**
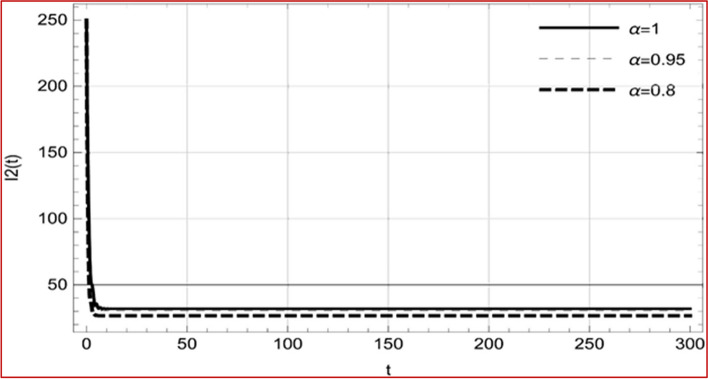
Memory profile of $I_{2} \left( t \right) \in \Gamma_{2}$ of system (4), for parameters describe in set 1 of Table 1 at $\tau_{1} = 1.375,\tau_{2} = 2.5$ and $u = { 0}$ for $\alpha = 0.8,\;0.95,\;1$ and $t \in \left[ {0,300} \right]$

**Fig. 31:**
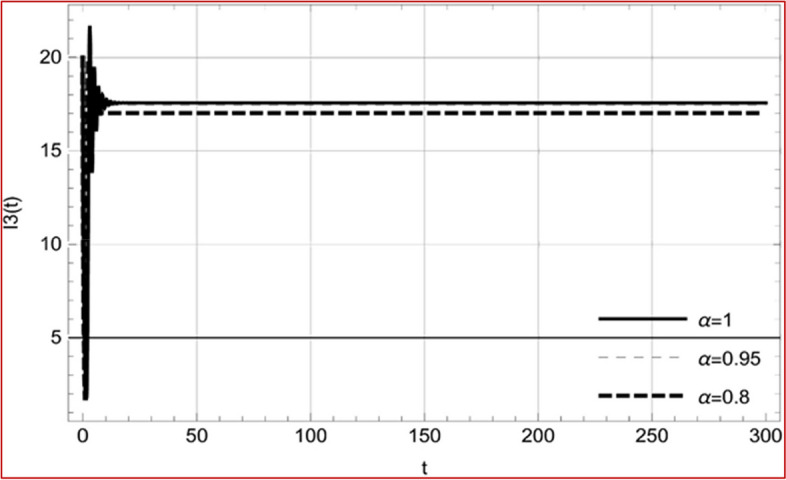
Memory profile of $I_{3} \left( t \right) \in \Gamma_{2}$ of system (4), for parameters describe in set 1 of Table 1 at $\tau_{1} = 1.375,\tau_{2} = 2.5$ and $u = { 0}$ for $\alpha = 0.8,\;0.95,\;1$ and $t \in \left[ {0,300} \right]$

**Fig. 32:**
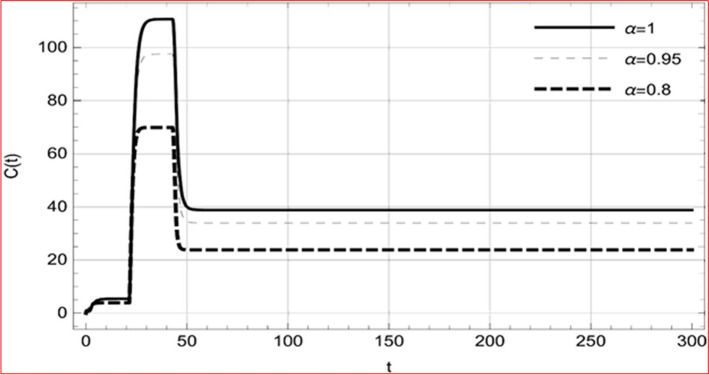
Memory profile of $Q\left( t \right) \in \Gamma_{2}$ of system (4), for parameters describe in set 1 of Table 1 at $\tau_{1} = 1.375,\tau_{2} = 2.5$ and $u = { 0}$ for $\alpha = 0.8,\;0.95,\;1$ and $t \in \left[ {0,300} \right]$

**Fig. 33:**
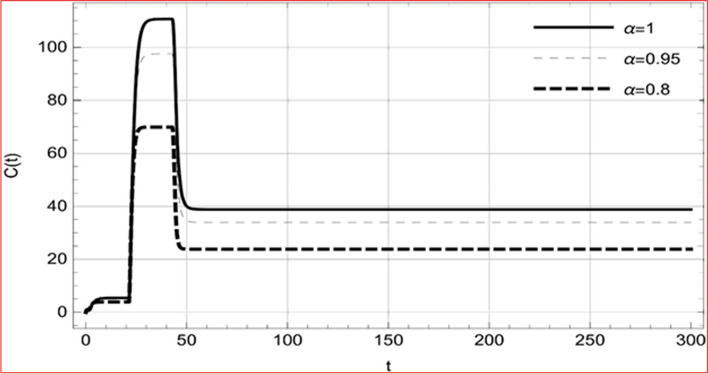
Memory profile of $C\left( t \right) \in \Gamma_{2}$ of system (4), for parameters describe in set 1 of Table 1 at $\tau_{1} = 1.375,\tau_{2} = 2.5$ and $u = { 0}$ for $\alpha = 0.8,\;0.95,\;1$ and $t \in \left[ {0,300} \right]$

**Fig. 34:**
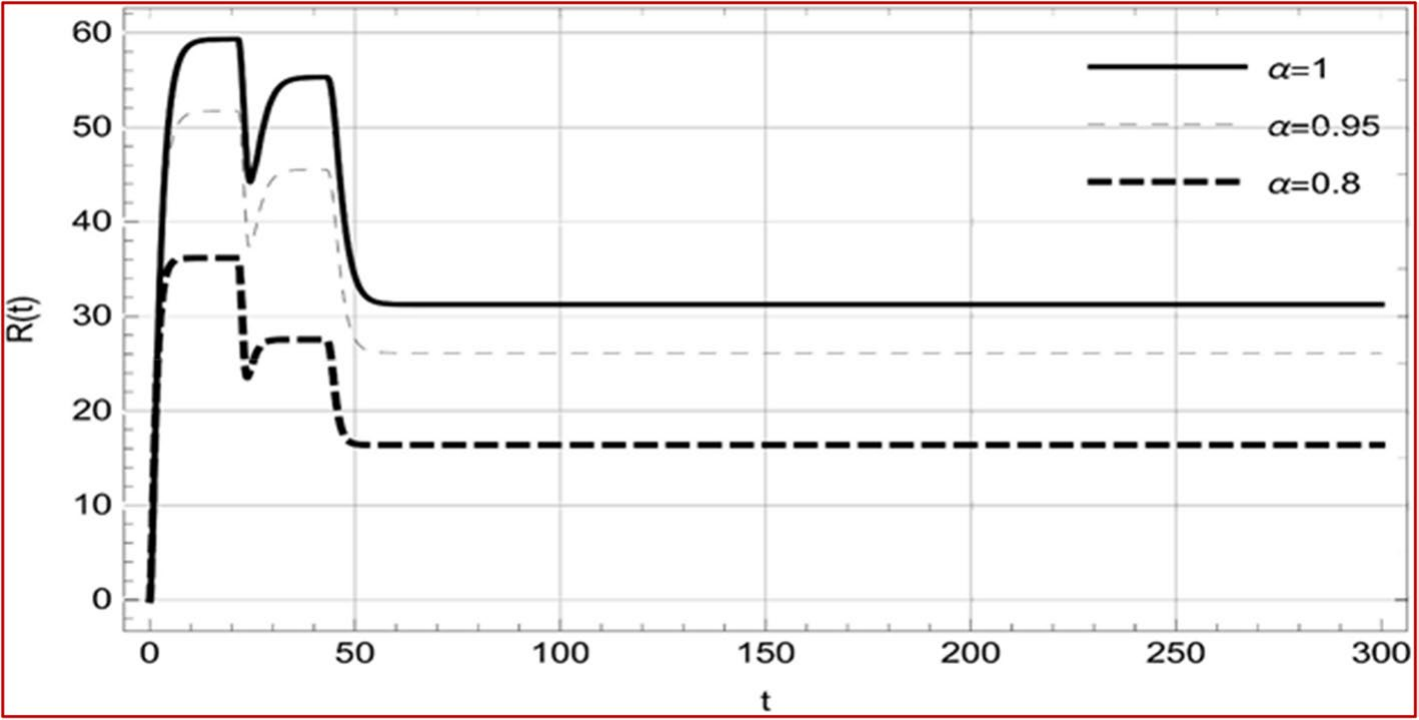
Memory profile of $R\left( t \right) \in \Gamma_{2}$ of system (4), for parameters describe in set 1 of Table 1 at $\tau_{1} = 1.375,\tau_{2} = 2.5$ and $u = { 0}$ for $\alpha = 0.8,\;0.95,\;1$ and $t \in \left[ {0,300} \right]$

On the other hand with set 2 of Table 1 at different fractional orders, the reproduction number and constant solutions are computed in Table 2, by setting the controlling parameter $u = 0$ and distinct values of transmission rate $\beta_{1} ,\beta_{2}$ and $\beta_{3}$. Consequently, the reproduction number $\text R_{\text o}$ is greater than 1, and the whole population contributes to the transmission of the disease. As a result, the number of infected patients increases, and the virus survives in the population due to the endemic nature of it. Table 3. presents the basic reproduction number $\text{R}_\text{o}$ and constant solutions at the disease-free state of system (4) for set 1 values of Table 1, with u = 1, at various values of $\alpha$. A pictorial representation of the endemic state of solutions of system (4) is presented in Figs. 19, 20, 21, 22, 23, 24, 25 and 26, at different fractional orders and time $t \in \left[ {0,40} \right]$ without delay. Additionally, we obtain the critical hopf bifurcation points $\tau_{1} = 1.375$ and $\tau_{2} = 2.5$ for the parameters described in set 2 of Table 1 for there is a theoretical statement in the following theorem (8–10) that the endemic state can be maintained if the critical points $\tau_{1} = 1.375$ and $\tau_{2} = 2.5$ and becomes unstable if $\tau_{1}> 1.375$ and $\tau_{2}> 2.5$, from the Figs. 27, 28, 29, 30, 31, 32, 33 and 34. There is a tendency for the equilibrium state of an endemic to become more unstable as more time elapses over the critical points.

**Table 2:** Presents the Basic Reproduction Number $\text{R}_\text{o}$ and Constant Solutions at the Endemic State of system (4) for Set 2 values of Table 1, with u = 0, at various values of $\alpha$

$\alpha$	$\text{R}_\text{o}$	$S\left( t \right)$	$E\left( t \right)$	$I_{1} \left( t \right)$	$I_{2} \left( t \right)$	$I_{3} \left( t \right)$	$Q\left( t \right)$	$C\left( t \right)$	$R\left( t \right)$
0.4	4.4334	23.537	15.163	25.5031	19.8722	15.5095	19.3245	11.2436	6.4047
0.5	4.4860	21.712	12.315	26.3094	21.2903	15.9355	21.1952	13.3129	7.8815
0.6	5.5492	19.915	17.585	27.0079	22.8613	16.3301	23.2911	15.9283	9.8479
0.7	5.6261	17.120	21.036	27.8072	24.6302	17.0203	25.6583	19.3081	12.5456
0.8	6.7211	15.312	92.728	28.4678	26.6601	17.3125	28.3611	23.7976	16.3825
0.9	7.8407	13.560	82.714	29.1430	29.0413	17.5655	31.4862	29.9625	22.1204
1	8.9945	12.712	71.037	29.7452	31.9110	17.8755	35.1538	38.8012	31.2366

**Table 3:** Presents the Basic Reproduction Number $\text{R}_\text{o}$ and Constant Solutions at the Disease-Free State of system (4) for Set 1 values of Table 1, with u = 1, at various values of $\alpha$

$\alpha$	$\text{R}_\text{o}$	$S\left( t \right)$	$E\left( t \right)$	$I_{1} \left( t \right)$	$I_{2} \left( t \right)$	$I_{3} \left( t \right)$	$Q(t)$	$C\left( t \right)$	$R(t)$
0.4	0.4334	298.537	0	0	0	0	0	0	0
0.5	0.4860	298.712	0	0	0	0	0	0	0
0.6	0.5492	298.915	0	0	0	0	0	0	0
0.7	0.6261	299.120	0	0	0	0	0	0	0
0.8	0.7211	299.312	0	0	0	0	0	0	0
0.9	0.8407	299.560	0	0	0	0	0	0	0
1	0.9945	299.712	0	0	0	0	0	0	0

**Fig. 35:**
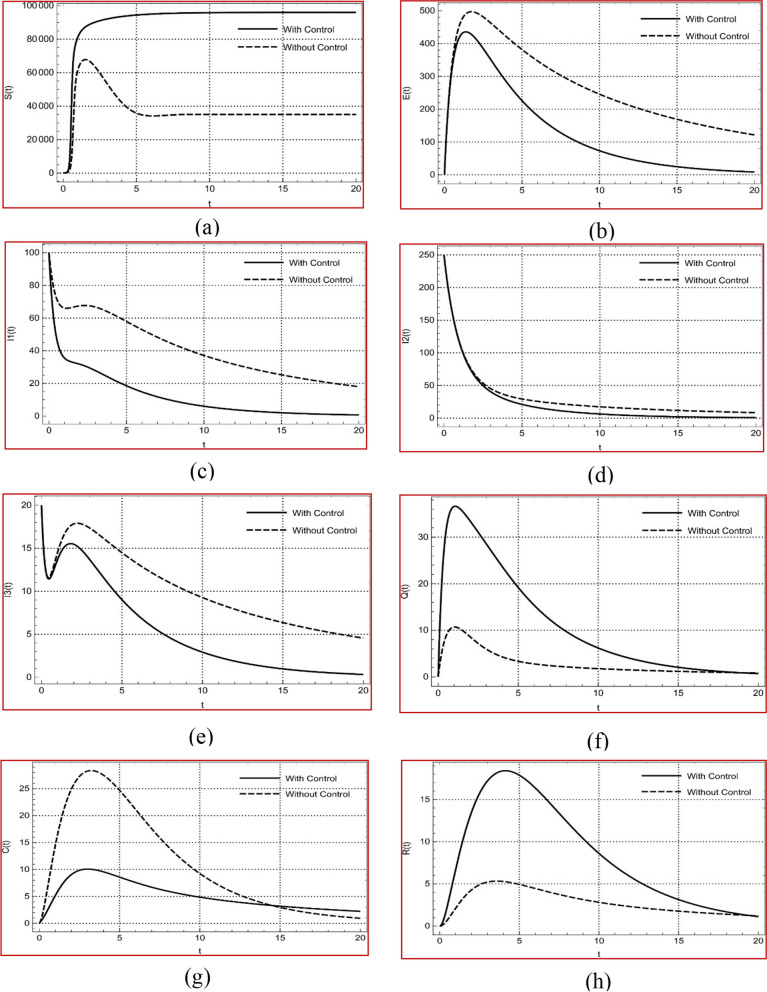
Impact of control interventions on state variables of system (4). The higher control strategy significantly reduce the disease burden over time

Figure 35 demonstrate the significance of control function $u$ in the simulations of state variable of system (4). It highlights critical role in reducing the disease burden within the population. The increase in awareness program shows a tremendous rise in the reduction of infected individuals and simultaneously a higher percent of the population moves towards the recovered compartments.

### Data fitting for estimation and comparison

The reduction of transmission of any pandemic disease are much dependent on it that weather the people are aware by it or not. For numerical manifestation presented for any infectious disease that fit into this compartmental model. We take recently pandemic disease as an example to relate it by our model. We chose as an example of the cumulative cases affecting Shanghai, China, from March 1, 2022, of COVID-19 under control measures. As a sample, we collected data from the website of Shanghai from March 1, 2022 to April 19, 2022 regarding mild and severe cases [45, 46]. It can be seen in Fig. 2 that there has been a rapid increase in confirmed cases of COVID-19, indicating that although people were aware of precautions when the epidemic began, they are not sufficient if preventive measures such as vaccines, treatments, or other non-pharmaceutical strategies are not taken as soon as possible, A widespread infection can quickly spread, putting the population at risk. A critical aspect of the validation of model is the comparison with the statistical data to estimate the parameters, by setting some of the selected parameters from literature [5, 7, 42] we considered four as being estimated by the nonlinear least square method to fit the statistical data [45] scaled to $1 \times 10^{6}$. Table 1 shows that the fitted parameters are in good agreement with the reported values. This indicates that our model is reliable and accurate. In order to calculate relative error, we use the formula below:

$$\frac{1}{10}\sum\limits_{i = 1}^{10} {\left| {\frac{{X_{i}^{real} - X_{i}^{approximate} }}{{X_{i}^{real} }}} \right|}$$

**Fig. 36:**
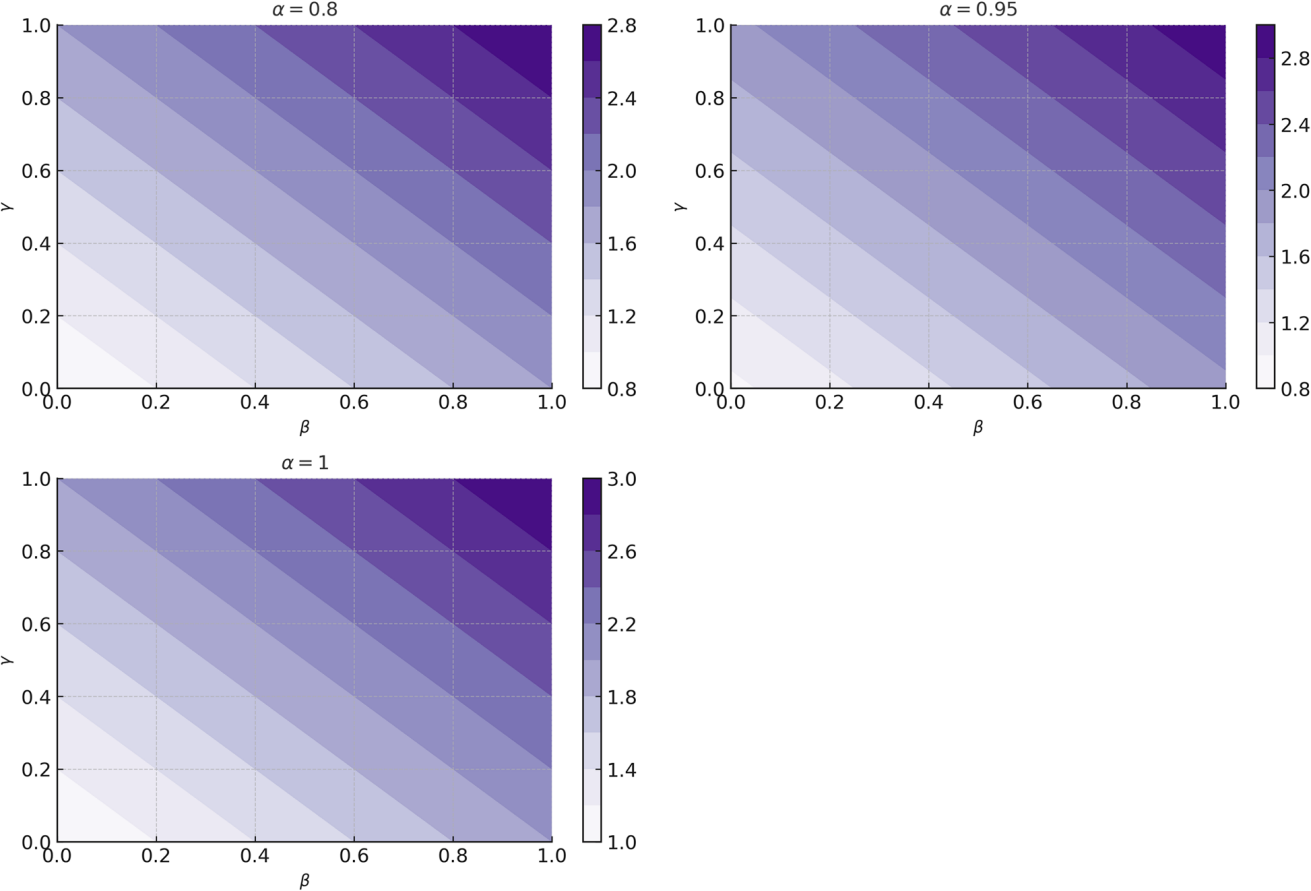
The sensitivity inspection of basic reproductive number for two parameters of Table 2. The lines of the contour plot represent the impact of model parameters on $\text{R}_\text{o}$ at distinct fractional order $\alpha = 0.8,\;0.95,\;1$

From Fig. 2, the simulation results shows less error with reported cases of Covid-19. The simulated results becomes more reliable and it can be used to analyze the trend of disease under control strategies. In order to develop pandemic control measures, It is imperative to conduct a sensitivity analysis of the parameters within the dynamical system. To determine which parameters will play a critical role in the analysis of control measures and reduction of basic reproduction numbers, we used the method in [42]. Figure 36 shows that the variation of the basic reproduction number with respect to some parameters describe in Table 1. Furthermore, In Table 4, we observe that the contact rates $\beta_{1} ,\beta_{2} ,\beta_{3}$ and the controlling parameter $u$ have higher influence on the basic reproduction number $\text{R}_\text{o}$ and the cost function $J$. These outcomes through sensitivity analysis is achieved by distinct fractional orders and time lag of $\tau_{1} = 1.375$ and $\tau_{2} = 2.5$. In this table the values of cost function $J$ and reproduction number shows that asymptomatic individuals with high transmission rates have the highest reproduction rate $\text{R}_\text{o}$ i.e. 0.7530 to 2.0567.due to the absence of awareness campaigns. Moreover, cost function $J$ against the population weights $\vartheta_{3} = 600$,$\vartheta_{2} = 300$, $\vartheta_{3} = 150$ and $\Phi = 100$. Which rapidly increase from $1.484 \times 10^{23}$ to $2.9784 \times 10^{23}$, at $\alpha = \;0.95$.

**Table 4:** Sensitivity of Basic Reproduction number $\text{R}_\text{o}$ and optimal inspection J based on transmission parameters and awareness scenario for the weight functions,$\vartheta_{1} = 100$ $\vartheta_{2} = 250$,$\vartheta_{3} = 400$ and $\Phi = \frac{1}{2}$, and parameter values from Table 1. For $\tau_{1} = 1.5$ and $\tau_{2} = 2.5$ at different values of $\alpha$

$\alpha$	$u$	$\beta_{1}$	$\beta_{2}$	$\beta_{3}$	$\text{R}_\text{o}$	$J$
0.95	0.6782	0.3781	0.01781	0.0378	0.7530	$1.484 \times 10^{23}$
	0.6782	0.432	0.01781	0.0378	0.8530	$1.9784 \times 10^{23}$
	0.6782	0.3781	0.03124	0.0378	1.0342	$2.0078 \times 10^{23}$
	0	0.3781	0.01781	0.0424	2.0567	$2.9784 \times 10^{23}$
1	0.6782	0.3781	0.01781	0.0378	0.7530	$1.484 \times 10^{23}$
	0.6782	0.432	0.01781	0.0378	0.8530	$1.9784 \times 10^{23}$
	0.6782	0.3781	0.03124	0.0378	1.0342	$2.0078 \times 10^{23}$
	0	0.3781	0.01781	0.0424	2.0567	$2.9784 \times 10^{23}$

## Important discussion about the model

### Model formulation and assumptions

- **Population Stratification**: The model stratifies the total population $N\left( t \right)$ into eight compartments: susceptible (S), exposed (E), pre-symptomatic infectious $I_{1} \left( t \right)$, mildly symptomatic infectious ($I_{2} \left( t \right)$), severely symptomatic infectious ($I_{3} \left( t \right)$), quarantined (Q), ICU (C), and recovered (R).
- **Transmission Pathways**: The flow between compartments is governed by transmission rates (βij​) that vary based on contact rates and progression of disease stages.
- **Delay Factors**: Delay terms (τi​) represent the incubation period and time before transitioning between compartments, improving the realism of disease dynamics.
- **Fractional Calculus**: The model incorporates fractional derivatives with order α\alphaα to account for memory effects, reflecting the influence of past infections on current disease states.
- **Control Measures**: Quarantine and awareness campaigns are modeled as dynamic control functions influencing transitions from $I_{1} \left( t \right)$​ to Q, $I_{2} \left( t \right)$ to Q, and S to Q.

### Parameter estimation

- **Data Source**: Parameters were estimated using COVID-19 case data from Shanghai (March–April 2022). A nonlinear least squares fitting was applied to minimize the discrepancy between simulated and reported data.
- **Initial Conditions**: Population values for each compartment were initialized based on real-world estimates (e.g., susceptible = 100, exposed = 0).
- **Sensitivity Analysis**: Parameters like β, quarantine rates, and awareness effectiveness were varied to assess their impact on the basic reproduction number $\text{R}_\text{o}$​ and disease progression.

### Numerical implementation

- **Software**: The model equations were solved using Mathematica 12.1, leveraging its built-in Runge–Kutta solver for delay differential equations.
- **Simulation Scenarios**: Two sets of parameters (Table 1) were used to simulate both disease-free and endemic scenarios under varying control intensities.
- **Reproduction Number**$\text{R}_\text{o}$: Derived analytically and validated through numerical simulations, $\text{R}_\text{o}$​ serves as a threshold for stability analysis.

### Stability analysis

- **Equilibrium Points**: Disease-free equilibrium (DFE) and endemic equilibrium (EE) conditions were derived mathematically.
- **Hopf Bifurcation**: Stability conditions were tested for oscillatory behaviors introduced by delay terms, providing insights into potential outbreak cycles.

### Optimal control problem

- **Cost Function**: The cost function (JJJ) incorporates the financial implications of quarantine and awareness efforts alongside the health burden of infections.
- **Pontryagin’s Maximum Principle**: Applied to derive adjoint equations and determine optimal control trajectories for interventions.
- **Control Profiles**: Simulations tested various combinations of quarantine and awareness strategies, highlighting their effects on $\text{R}_\text{o}$​and infection dynamics.

### Validation and sensitivity analysis

- **Validation**: The model outputs were validated against real-world COVID-19 data, achieving close alignment with observed trends in infection peaks and recovery rates.
- **Sensitivity Analysis**: Parameters influencing $\text{R}_\text{o}$​, such as contact rates and awareness efficiency, were varied to evaluate their significance.

### Outputs and visualization

**Figures and Tables**: Graphs illustrate compartment dynamics over time, memory effects of fractional derivatives, and the impact of control measures. Sensitivity results and reproduction number variations are summarized in contour plots and tabular data.

## Conclusion

In conclusion, the SEI1I2I3QCR model provides a comprehensive framework for understanding the dynamics of COVID-19 transmission and evaluating the effectiveness of non-pharmaceutical interventions. By incorporating fractional-order delay differential equations and differentiating among various stages of infection—specifically asymptomatic, mild, and severe cases—the model captures the complexities of disease progression more accurately than traditional models. Our findings demonstrate the critical role of symptom awareness and timely interventions, such as quarantine and awareness campaigns, in reducing transmission rates and achieving disease control. The sensitivity analysis further highlights the importance of parameters such as transmission rates and compliance levels in influencing the basic reproduction number (R0), guiding effective public health strategies.

Moreover, the formulation of the optimal control problem reveals that strategically implemented control measures can significantly minimize both health and economic costs associated with the pandemic. By demonstrating the effectiveness of adaptive intervention strategies, the model not only informs current public health responses but also lays the groundwork for future epidemic management across diverse infectious diseases. As we move forward, the insights gained from this study underscore the necessity for a flexible, data-driven approach to public health interventions, enabling us to respond effectively to ongoing and emerging infectious disease threats. The SEI1I2I3QCR model thus stands as a valuable tool for researchers and policymakers alike, offering robust methodologies for optimizing epidemic control efforts in an increasingly complex global health landscape.

This paper depicted the Spreading of Infectious disease in any society or country of the world. To assess the community wide impact of control measure and mitigation strategies from different source like Television, Newspaper and social media etc. which is serious measure for public health units to assess the population and collect accurate data of infected individuals. Therefore, the control measure in the asymptomatic class was introduced in the epidemic model to provide the awareness to the population and get a more accurate decisions about the disease mitigation. In this study we use the non-pharmaceutical intervention in our model in order to study the dynamics of infectious disease that could fit in our model. We develop the fractional order mathematical model and after evaluating some mathematical analysis including positivity and stability results of equilibrium point. We analyzed its dynamic properties and by considering the control measure an optimal cost function will establish. In this study, numerical simulation supported the stability results and sensitivity analysis of the basic reproduction number, which were presented in the form of tables and graphs. As a result of the entire study, the following facts and figures are obtained:

- The Strategy of using awareness campaign in asymptomatic class are significant attempt to aware individuals about disease and move to quarantine class.
- Implementation of control variable and less force of infection rate of disease the systematic reading of each compartment is studied with fractional derivative effects.
- Due to the absence of awareness in the population disease will not die out and will endemically survive as the population becomes deceit transmitter of infectious disease
- The strong impact of controlling parameter on the dynamical behavior of basic reproduction number with memory effects are carried out.
- Implementation of measures to control the transmission risk of a disease while optimizing costs and considering the time lag, which may lead to individuals requiring intensive care unit (ICU) treatment

The proposed work can be applied to understanding disease patterns that are transmitted between humans through contact and to devising effective control strategies to cure these diseases. The successful investigations show the aim of the work that the use of non-pharmaceutical interventions played a significant role in non-aware individuals at early stages of disease spread to reduce the number of infective individuals and minimize the total cost associated with it. Our future goal is to cover characteristics of society that are invisible causes of disease transmission between humans, animals, and plants.

## Data Availability

Data will be available on request by contacting the corresponding author OR via the author M. Ijaz Khan 2106391391@pku.edu.c

## Appendix

$$\begin{array}{lllll} T_{4} = { - }\left( {a_{33} + a_{55} + a_{7722} + a_{{{33}}} + a_{{{44}}} + 2a_{{{55}}} + a_{{{23}}} + a_{{{34}}} } \right) \hfill \\ T_{3} = \, \left( {a_{22} a_{33} + a_{22} a_{44} + a_{22} a_{55} + a_{22} a_{55} + a_{33} a_{66} + a_{33} a_{55} + a_{44} a_{66} } \right) \hfill \\ T_{2} = \, \left( {a_{22} a_{33} a_{44} + a_{22} a_{33} a_{55} + a_{22} a_{44} a_{55} + a_{33} a_{44} a_{55} + a_{33} a_{44} a_{66} } \right) \hfill \\ T_{1} = \, \left( {a_{22} a_{33} a_{44} a_{55} + a_{22} a_{33} a_{44} a_{66} + a_{22} a_{33} a_{55} a_{77} + a_{22} a_{33} a_{44} a_{88} + a_{22} a_{33} a_{44} a_{55} } \right) \hfill \\ T_{0} = \, \left( {a_{22} a_{33} a_{44} a_{55} a_{66} + a_{22} a_{33} a_{55} a_{66} a_{77} + a_{22} a_{33} a_{44} a_{77} a_{88} + a_{22} a_{33} a_{44} a_{55} a_{66} } \right) \hfill \\ U_{3} = a_{{{25}}} a_{33} a_{44} a_{52} + a_{{{24}}} a_{{{33}}} a_{{{42}}} + a_{23} a_{{{32}}} a_{{{44}}} - a_{{{23}}} a_{{{33}}} a_{{{44}}} \hfill \\ U_{2} = a_{{{25}}} a_{33} a_{44} + a_{{{24}}} a_{{{33}}} a_{22} + a_{23} a_{{{33}}} a_{{{44}}} - a_{{{13}}} a_{{{33}}} a_{{{44}}} \hfill \\ U_{1} = a_{{{24}}} a_{44} a_{55} + a_{{{24}}} a_{{{33}}} a_{{{44}}} + a_{23} a_{{{32}}} a_{66} - a_{{{23}}} a_{{{33}}} a_{77} \hfill \\ U_{0} = a_{{{22}}} + a_{33} + a_{44} + a_{55} + a_{66} + a_{77} + a_{{{23}}} a_{{{33}}} a_{{{44}}} \hfill \\ V_{2} = a_{{{24}}} a_{{{44}}} a_{{{33}}} + a_{{{34}}} a_{44} a_{55} + a_{52} a_{{{44}}} a_{{{77}}} \hfill \\ V_{1} = a_{{{23}}} a_{{{42}}} + a_{{{34}}} a_{43} b_{63} + a_{32} a_{{{44}}} b_{{{76}}} \hfill \\ V_{0} = a_{{{23}}} a_{{{42}}} + a_{{{34}}} a_{43} b_{63} + a_{32} a_{{{44}}} b_{{{76}}} \hfill \\ W_{1} = b_{{{23}}} c_{86} + a_{43} b_{63} \hfill \\ W_{0} = c_{86} c_{87} + b_{76} \hfill \\ \end{array}$$
